# Modulation of the unfolded protein response with a C-terminal fragment of MANF facilitates recovery in models of multiple sclerosis

**DOI:** 10.1016/j.ymthe.2025.10.023

**Published:** 2025-10-11

**Authors:** Tapani K. Koppinen, Carolina R. Reyes, Jinhan Nam, Aastha Singh, Shibajee Mandal, Liam Beckett, Alba Montedeoca, Tuomas A.E. Kallionpää, Maria Lindahl, Francisco J. Rivera, Merja H. Voutilainen

**Affiliations:** 1 Division of Pharmacology and Pharmacotherapy, Faculty of Pharmacy, University of Helsinki, Helsinki, Finland; 2 Translational Regenerative Neurobiology Group (TReN), Molecular and Integrative Biosciences Research Programme (MIBS), Faculty of Biological and Environmental Sciences, University of Helsinki, Helsinki, Finland; 3 Institute of Biotechnology, HiLIFE, University of Helsinki, Helsinki, Finland

**Keywords:** remyelination, neuroinflammation, unfolded protein response, ER homeostasis, multiple sclerosis, proteostasis

## Abstract

Inflammation in multiple sclerosis leads to chronic activation of a cellular stress mechanism, the unfolded protein response (UPR), which is thought to both exacerbate neuroinflammation and prevent regenerative tissue responses such as remyelination. The UPR-modulating protein MANF has shown great promise for attenuating chronic UPR activation and enhancing tissue regeneration in various disease models but does not reach the CNS when given peripherally. We utilized C-MANF, a C-terminal fragment of MANF, and showed that subcutaneous administration of C-MANF promoted motor function recovery and tissue regeneration in a mouse model of autoimmune demyelination. We demonstrated that C-MANF suppresses neuroinflammatory activation and facilitates the recovery of oligodendrocytes after demyelination, while reducing long-term activation of the UPR. Furthermore, we showed that C-MANF enhances myelination of primary OPCs in culture, that promotion of remyelination in cerebellar organotypic slice cultures is dependent on UPR-modulation, and that exogenously applied C-MANF suppresses chronic activation of all three UPR pathways in oligodendroglia. Finally, we showed that demyelination in MANF-deficient brains leads to extensive neuroinflammation and CNS degeneration, implicating UPR modulation by MANF as a key component in tissue responses to demyelination. Altogether, we show that UPR modulation with C-MANF is a promising new therapeutic approach for treating neuroinflammatory demyelination.

## Graphical abstract

**Figure:**
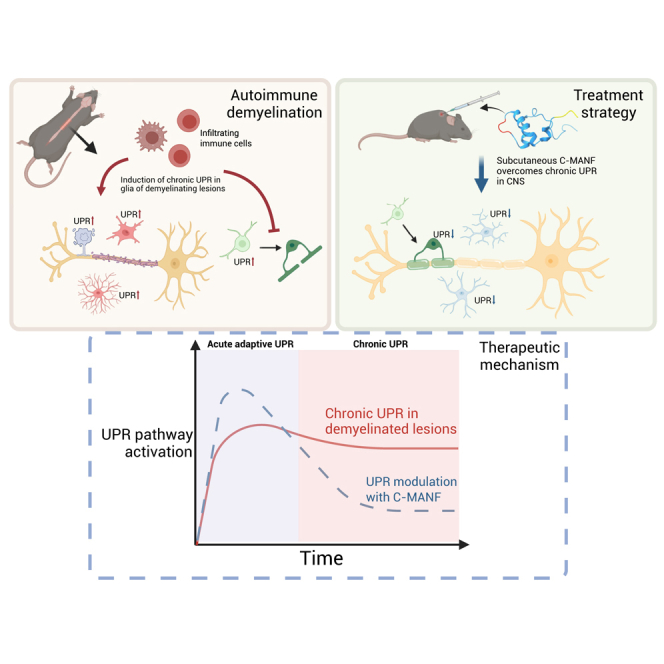

## Teaser

Autoimmune neuroinflammation in diseases such as multiple sclerosis induces activation of a stress pathway called the unfolded protein response (UPR) in demyelinated lesions, preventing remyelination. C-MANF, a novel protein fragment, enhanced lesion regeneration after subcutaneous injection by inhibiting chronic UPR activation. This reduced neuroinflammation and enhanced remyelination.

## Introduction

Multiple sclerosis (MS) is a complex and chronic disease, characterized by autoimmune-mediated demyelination of the central nervous system (CNS).^1^^,^^2^ While the number and efficacy of available disease-modifying treatments have increased in the past decade, most recently with the introduction of anti-CD20 B cell-depleting therapeutics, all of these treatments target peripherally driven inflammatory processes. Thus, there is an acute need for treatments capable of efficiently preventing the progression of neurological pathology and improving tissue repair. One of the key factors underlying MS progression is the weakened ability of the CNS to conduct remyelination, the process of forming new myelin around axons.^3^

Remyelination requires the coordination of differentiating oligodendrocyte precursor cells (OPCs) with surrounding cells, particularly microglia^4^^,^^5^^,^^6^^,^^7^ and astrocytes.^8^^,^^9^^,^^10^^,^^11^^,^^12^ In MS lesions, which feature strong and chronic neuroinflammatory activation, the tissue microenvironment created by astrocytes and microglia becomes hostile to OPC differentiation.^12^^,^^13^^,^^14^ Potential novel therapeutic approaches to improve CNS regeneration in MS include inducing OPC differentiation,^15^^,^^16^^,^^17^^,^^18^ protecting mature oligodendrocytes against apoptosis,^19^ and inhibiting glial neuroinflammatory responses,^20^^,^^21^^,^^22^^,^^23^^,^^24^ but addressing only one component of the pathological cascade featured in MS lesions may be an ineffective approach.

The unfolded protein response (UPR) is an adaptive cellular stress response to the accumulation of unfolded or misfolded proteins in the endoplasmic reticulum (ER).^25^ UPR activation aims to restore ER homeostasis by increasing protein-folding capacity via three signaling cascades operating in parallel: inositol-requiring protein-1 (IRE1), protein kinase RNA-like ER kinase (PERK), and activating transcription factor 6 (ATF6). These pathways attempt to reduce ER protein folding load by increasing proteostasis and enhancing protein degradation, while simultaneously increasing folding capacity by upregulating the production of chaperones and genes involved in antioxidant defense.^25^^,^^26^ Chronic sustained activation of the UPR, however, shifts the goals of the UPR from adaptive cytoprotection toward proapoptotic and proinflammatory signaling^27^^,^^28^^,^^29^^,^^30^ as an evolutionary response to intracellular infection. It has been implicated as a key driver of neuroinflammation^12^^,^^31^ and is a major factor behind oligodendrocyte and neuron apoptosis.^32^^,^^33^ Chronic UPR activation is also a shared pathological feature of neurodegenerative diseases and features prominently in the brains of MS patients.^12^^,^^34^^,^^35^^,^^36^^,^^37^

One way by which the UPR attempts to restore homeostasis is through the upregulation of MANF (mesencephalic astrocyte-derived neurotrophic factor), an ER-localized protein with anti-apoptotic properties that is highly expressed in tissues with high metabolic loads, such as the liver, testes, and pancreas.^38^^,^^39^^,^^40^^,^^41^ MANF is uniquely capable of restoring metabolic homeostasis and inducing regeneration in damaged tissue^42^^,^^43^^,^^44^^,^^45^^,^^46^^,^^47^^,^^48^^,^^49^ via UPR modulation,^50^^,^^51^ although it has also been suggested to reduce inflammation via UPR-independent nuclear factor κB (NF-κB) suppression.^46^^,^^52^^,^^53^ This makes it a highly promising candidate for regenerative therapy, but its potential in treating neurological diseases such as MS is restricted by its poor bioavailability in the brain after peripheral administration.^49^^,^^54^

Recent breakthroughs with MANF’s sibling protein CDNF (cerebral dopaminergic neurotrophic factor) have shown that a shorter CDNF fragment restricted to only regions of the C-terminal domain is sufficient to prevent the degeneration of dopaminergic neurons in models of Parkinson disease and amyotrophic lateral sclerosis (ALS), while exhibiting drastically improved blood-brain barrier (BBB) penetration.^55^^,^^56^ Like CDNF, the C-terminal domain of MANF features an ER retention signal and a CXXC motif.^57^^,^^58^^,^^59^ Similarly, C-MANF (the C-terminal fragment of MANF) potently protects cultured neurons against cellular stress and interacts with all three UPR sensors at high affinity.^51^^,^^57^ As C-MANF shares the properties that make MANF neuroprotective and neurorestorative, while possibly featuring better bioavailability in the brain due to its reduced size, we hypothesize that it could function as a systemically administered treatment for MS, which could utilize UPR modulation to promote oligodendrocyte survival and differentiation while suppressing neuroinflammation.

In this work, we showed that C-MANF is neuroprotective in several disease models of MS. Subcutaneous administration of C-MANF promoted recovery in an experimental autoimmune encephalomyelitis (EAE) mouse model, while reducing demyelination and microglial and astrocytic activation. C-MANF boosts OPC differentiation *in vitro*, and when applied directly to organotypic cerebellar slices, it enhances remyelination in a dose- and UPR-dependent manner. In cultured oligodendroglial cells, C-MANF increases acute UPR pathway activation, while reducing prolonged activation of the UPR pathways after toxin-induced ER stress. Finally, although mice lacking endogenous MANF expression exhibit normal myelination, they were unable to resolve toxin-induced demyelination. Thus, MANF is a key regulator of tissue responses to demyelination, and C-MANF represents a potential UPR-modulating treatment for MS.

## Results

### Subcutaneously administered C-MANF alleviates disease progression in a model of experimental autoimmunity

**Figure 1:**
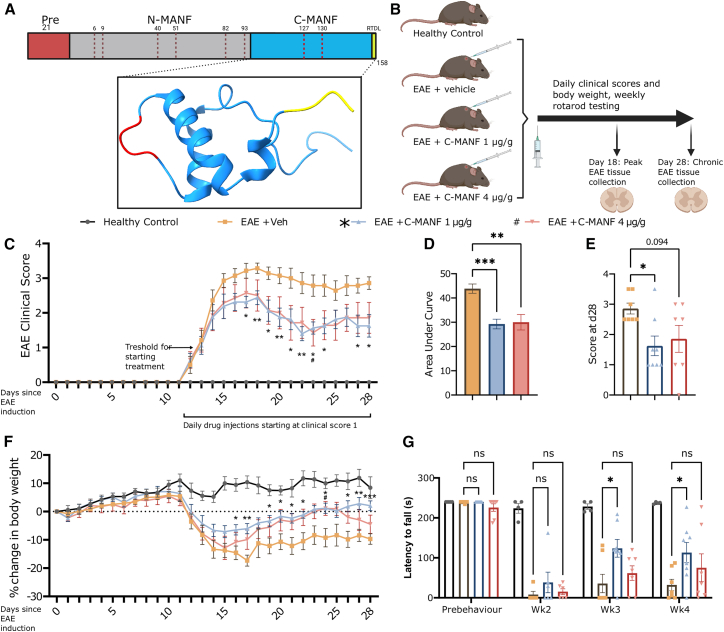
Subcutaneous C-MANF alleviates EAE progression (A) The structure of C-MANF and relationship with hMANF. NMR structure of C-MANF adapted from Hellman et al.^58^ (PDB: 2KVE; image created with ChimeraX 1.5). (B) Experimental outline and groups for testing of C-MANF in EAE. (C) Clinical scores were studied daily, and daily subcutaneous injections C-MANF (1 μg/g [*n* = 8] or 4 μg/g [*n* = 7]) or vehicle (*n* = 7) were administered to individual mice after they reached a score of 1. (D and E) (D) AUC (area under the curve) of group mean scores and (E) individual scores at the predetermined endpoint of day 28 after EAE induction. (F) Individual mice were weighed daily, and group average weights are reported as the percentage change in body weight relative to the day of EAE induction. (G) Motor coordination was tested using the rotarod apparatus before EAE induction and on days 14, 21, and 28 after EAE induction. Only mice experiencing EAE symptoms and rolled into treatment groups were included at postinduction time points. Lines and bar graphs represent group means ± SEMs, scatterplot represents individual myelinated axons with a simple linear regression ±95% confidence bands; ∗/#*p* ≤ 0.05; ∗∗*p* ≤ 0.01; ∗∗∗*p* ≤ 0.001; ns, not significant. (C and F) Matched two-way ANOVA, (D and E) one-way ANOVA, and (G) matched mixed effects (REML) model, all followed by Dunnett’s post hoc test. (A and B) Diagrams created in BioRender (https://BioRender.com/fww6ya0).

C-MANF was synthesized as a 63-amino acid (aa) protein with a CXXC motif and an RTDL ER retention site at the C terminus^58^ (Figure 1A). The EAE model recapitulates chronic autoimmune-mediated demyelination in rodents. We previously evaluated the efficacy of full-length human MANF in EAE mice by Nam et al.,^54^ finding that with peripheral delivery MANF has a limited capacity to preserve motor function in the early stage of the disease. To test whether C-MANF has improved pharmacokinetic properties, we studied the effects of peripherally delivered C-MANF on a therapeutic EAE treatment paradigm. After individual C57Bl/6JRcc mice began to show early symptoms of EAE, ascending paralysis starting with a limp tail, rolling enrollment was used to assign mice to balanced treatment groups. Mice received C-MANF at 1 or 4 μg/g or vehicle (PBS), as daily subcutaneous (s.c.) injections starting at clinical score 1 (Figure 1B). Motor coordination was analyzed with the rotarod test before EAE induction, then weekly starting on day 14 after EAE induction, and exploratory locomotion was assayed with the open field test before euthanasia at the endpoint of day 28.

Compared with vehicle-treated mice, mice that received C-MANF showed overall significantly ameliorated clinical symptoms, as calculated by the mean area under the curve of the clinical score (Figures 1C and 1D) (ANOVA F(2, 19) = 11.48; vehicle vs. 1 μg/g C-MANF *p* < 0.001; vehicle vs. 4 μg/g C-MANF *p* < 0.01), and the group receiving 1 μg/g C-MANF had a significantly lower average score at the endpoint of 28 days (Figure 1E) (ANOVA F(2, 19) = 3.796, *p* < 0.05). The reduction in clinical score was reflected in reduced body weight loss, as treatment with 1 μg/g C-MANF led to a significant recovery in body weight by day 28, reported as percentage change from starting body weight (Figure 1F; vehicle vs. 1 μg/g C-MANF *p* < 0.001). While the performance of all the EAE groups in the rotarod test displayed extreme motor deficit after EAE onset at week 2, the group receiving 1 μg/g C-MANF showed a significant improvement in motor coordination compared to the vehicle-treated group at weeks 3 and 4 (Figure 1G) (restricted maximum likelihood [REML] F(3, 22) = 20.55, *p* < 0.05 at both time points). Open field testing performed on day 28 indicated that treatment with 1 μg/g C-MANF caused a nonsignificant trend toward increased ambulation (Figures S1A–S1D).

### C-MANF prevents demyelination and axon loss during chronic EAE

**Figure 2:**
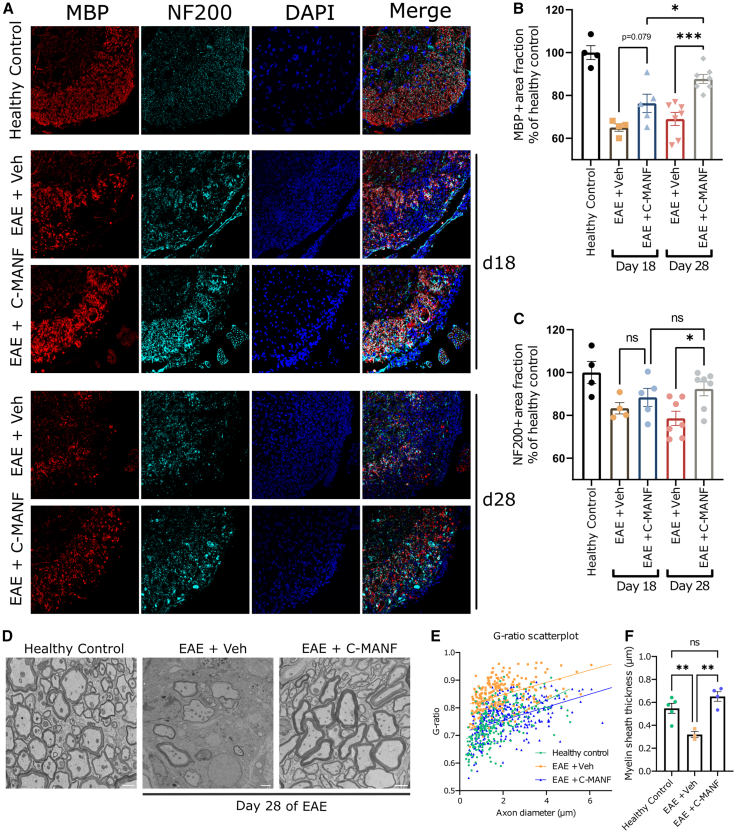
Neuroprotective effects of C-MANF in the EAE spinal cord (A–C) (A) Representative 20× magnification IF images stained for MBP, NF-200, and DAPI, taken from the analyzed area in the lumbar spinal cords of untreated healthy control mice and EAE animals euthanized at 18 or 28 days after EAE induction. Scale bar, 100 μm. Images were taken at three different sites in the ventral white matter and used to analyze (B) the MBP^+^ area fraction and (C) the NF-200^+^ area fraction in vehicle-treated and 1 μg/g C-MANF-treated EAE spinal cords, reported as percentage of the average of the healthy control group (pooled from day 18 and day 28, *n* = 4). (D) Representative TEM images of lumbar ventral spinal cord white matter of EAE mice treated with vehicle or 1 μg/g C-MANF. Scale bar, 1 μm. (E) Visualized G-ratio to axon diameter relationship, each dot representing a single myelinated axon (healthy control *n* = 250, EAE + vehicle *n* = 237, and EAE +1 μg/g C-MANF *n* = 300). (F) Mean thickness of myelin sheaths per EAE mouse spinal cord. Bar graphs represent group means ± SEMs; ∗*p* ≤ 0.05; ∗∗*p* ≤ 0.01; ∗∗∗*p* ≤ 0.001. (B, C, and F) One-way ANOVA followed by Šidak’s post hoc test.

To further analyze the neuroprotective effects of C-MANF on EAE mice, we euthanized mice treated with 1 μg/g C-MANF or vehicle at days 18 (peak phase) and 28 (chronic phase) after EAE induction. Immunohistochemical analyses of MBP (myelin basic protein) in the white matter of the lumbar spinal cord showed that while treatment with C-MANF did not inhibit myelin loss at the peak of the disease, myelination recovered and was significantly improved compared to that in the vehicle-treated groups at the end of the experiment (Figure 2B) (ANOVA F(4, 22) = 18.83; EAE + C-MANF day 18 vs. day 28 *p* < 0.05; day 28 EAE + C-MANF vs. EAE + vehicle *p* < 0.001), indicating that C-MANF increases myelin recovery in EAE mice.

Chronic EAE is known to feature progressive loss of axons in the spinal cord, which correlates with chronic disability, despite intermittent relapses and remissions in clinical score. This resembles the chronic axonal degeneration observed in MS patients, especially those suffering from progressive MS.^60^^,^^61^^,^^62^ We observed that treatment with C-MANF prevented the progressive axonal loss in the lumbar spinal cord (Figure 2C) (ANOVA F(4, 22) = 4.622; day 28 EAE + C-MANF vs. EAE + vehicle *p* < 0.05), as determined by staining for neurofilament heavy chain (NF-200).

Ultrastructural morphometry analysis was performed on transmission electron microscopy (TEM)-acquired micrographs using a plugin for ImageJ software.^63^ The spinal cords of 1 μg/g C-MANF-treated EAE mice exhibited an increased presence in myelinated axons compared to those of vehicle-treated mice (Figure 2D). Analysis of the G-ratio across axonal sizes revealed an increase in myelination (Figure 2E), a significant decrease in average myelin sheath thickness in EAE, and an increase in C-MANF-treated EAE spinal cords (Figure 2F) (ANOVA F(2, 9) = 13.79; healthy control vs. EAE + vehicle *p* < 0.01; EAE + vehicle *p* < 0.01), supporting the hypothesis that C-MANF rescues demyelination in EAE mice.

Western blot analysis of the extracted proteins indicated a positive non-significant trend in both MBP and NF light-chain levels. We also detected a significant decrease in astrocytic proinflammatory marker GFAP on day 28 (Figures S2A and S2B), suggesting that treatment with C-MANF was linked to alterations in astrogliosis.

### C-MANF reduces neuroinflammation and promotes oligodendrocyte recovery by regulating chronic UPR in EAE

**Figure 3:**
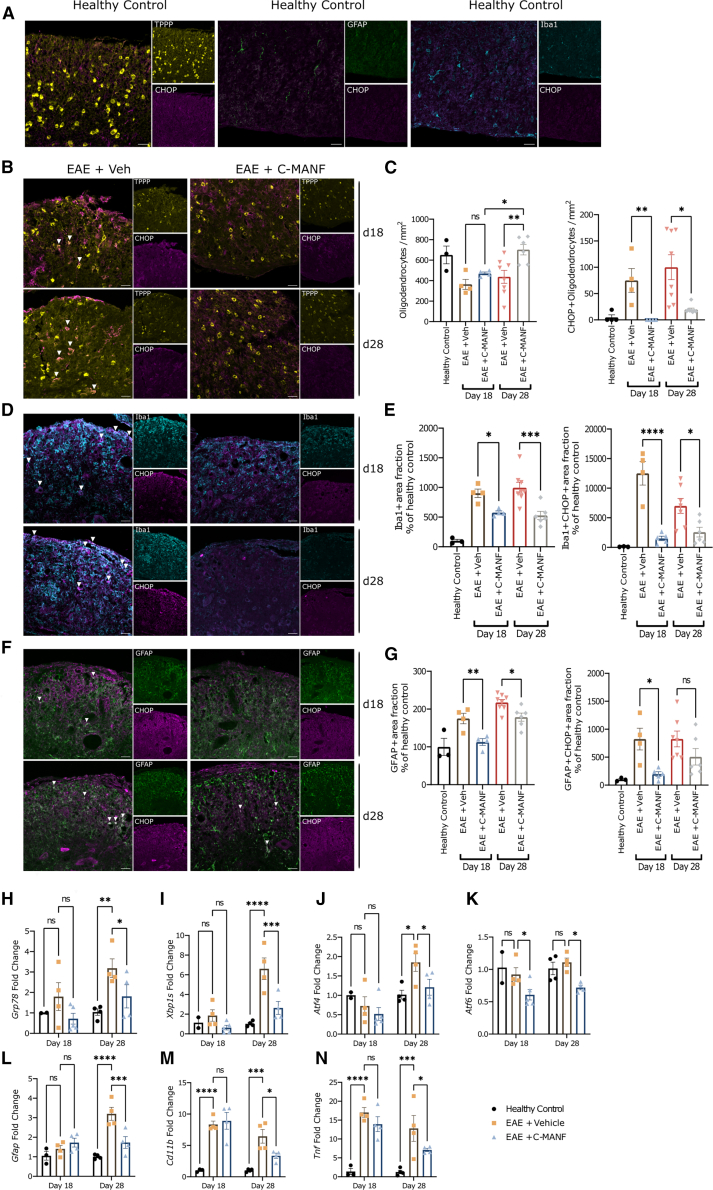
Chronic UPR suppression by C-MANF correlates with improved oligodendrocyte recovery and reduced neuroinflammation and astrogliosis in EAE (A) Representative IF images stained for TPPP (mature oligodendrocytes), GFAP (astrocyte marker), Iba1 (microglia marker), and CHOP (chronic proapoptotic UPR marker) in the lumbar spinal cord white matter of healthy control mice show a lack of CHOP expression in glia. (B) Representative IF images stained for TPPP and CHOP showing the presence of CHOP^+^ oligodendrocytes (denoted by white arrows) in EAE spinal cords. (C) Counts of TPPP^+^ oligodendrocytes and CHOP^+^TPPP^+^ oligodendrocytes normalized to analyzed spinal cord surface area. (D) Representative IF images stained for Iba1 and CHOP show the presence of CHOP^+^ microglia (denoted by white arrows) in EAE spinal cords. (E) Analyzed Iba1^+^ and Iba1^+^CHOP^+^ surface area fractions, reported as percentage of the average of the healthy control group. (F) Representative IF images stained for GFAP and CHOP show the presence of CHOP^+^ astrocytes (denoted by white arrows) in EAE spinal cords. (G) Analyzed GFAP^+^ and GFAP^+^CHOP^+^ surface area fractions, reported as percentage of the average of the healthy control group. For (C), (E), and (G), healthy controls were pooled from both time points, and EAE spinal cords were from days 18 or 28 after EAE induction. (H–N) mRNA extracted from EAE spinal cords was analyzed by qPCR for the expression of (H) *Grp78*, (I) *Xbp1s*, (J) *Atf4*, (K) *Atf6*, (L) *Gfap*, (M) *Cd11b*, and (N) *Tnfα* gene expression and reported as fold change in expression relative to time point-matched healthy controls. (A, B, D, and F) Scale bar, 25 μm. Bar graphs represent group means ± SEMs; ∗*p* ≤ 0.05; ∗∗*p* ≤ 0.01; ∗∗∗*p* ≤ 0.001; ∗∗∗∗*p* ≤ 0.0001. (C) First graph, one-way ANOVA, followed by Šidak’s post hoc test; second graph, Kruskal-Wallis test, followed by Dunn’s post hoc test. (E and G) One-way ANOVA, followed by Šidak’s post hoc test. (H–N) Two-way ANOVA, followed by Holm-Šidak’s post hoc test.

Both EAE and MS exhibit the strong activation of neuroinflammatory microglia and astrocytes expressing CHOP, a proapoptotic and proinflammatory UPR component upregulated by chronic activation of the IRE1α, PERK, and ATF6 pathways.^34^^,^^35^^,^^64^^,^^65^ Myelinating oligodendrocytes also exhibit CHOP activation in EAE spinal cords and acute MS lesions, which contributes to demyelination via oligodendrocyte apoptosis. We performed a set of immunofluorescence (IF) analyses of CHOP expression in TPPP^+^ myelinating oligodendrocytes, Iba1^+^ microglia, and GFAP^+^ astrocytes. Unlike in healthy control mice, EAE spinal cords had reduced oligodendrocyte counts and intense microgliosis and astrogliosis, with CHOP expression apparent in all three glial cell types (Figures 3A, 3B, 3D, and 3F). As observed with MBP staining earlier, treatment with C-MANF did not prevent oligodendrocyte loss at the peak of EAE, but it did lead to a recovery in cell counts by day 28 (Figure 3C) (ANOVA F(4, 20) = 5.478; EAE + vehicle vs. EAE + C-MANF day 28 *p* < 0.01; EAE + C-MANF day 18 vs. day 28 *p* < 0.05). Simultaneously, treatment with C-MANF drastically reduced the number of CHOP^+^ oligodendrocytes in the lumbar white matter (Figure 3C) (Kruskal-Wallis statistic = 20.87; day 18 EAE + C-MANF vs. day 18 EAE + vehicle *p* < 0.01; day 28 EAE + C-MANF vs. day 28 EAE + vehicle *p* < 0.05). Treatment with C-MANF reduced Iba1^+^ microgliosis at both time points (Figure 3E) (ANOVA F(4, 20) = 16.02; day 18 EAE + C-MANF vs. EAE + vehicle *p* < 0.05; day 28 EAE + C-MANF vs. day 28 EAE + vehicle *p* < 0.001), while also leading to a strong decrease in the fraction of CHOP^+^ microglia (Figure 3E) (ANOVA F(4, 20) = 15.28; EAE + vehicle vs. EAE + C-MANF day 18 *p* < 0.0001; day 28 EAE + C-MANF vs. day 28 EAE + vehicle *p* < 0.05). Treatment with C-MANF also reduced GFAP^+^ astrogliosis at both time points (Figure 3G) (ANOVA F(4, 20) = 17.68; EAE + vehicle vs. EAE + C-MANF day 18 *p* < 0.01; EAE + vehicle vs. EAE + C-MANF day 28 *p* < 0.05), while reducing the expression of CHOP in astrocytes on day 18 (Figure 3G) (ANOVA F(4, 20) = 5.207; EAE + vehicle vs. EAE + C-MANF day 18 *p* < 0.05).

Next, we performed qPCR to analyze changes in the expression of genes related to the UPR and neuroinflammation. EAE significantly increased the expression of the UPR genes *Grp78* (primary sensor of ER stress), *Xbp1s* (IRE1α pathway-activated transcription factor) and *Atf4* (PERK pathway-activated transcription factor) on day 28, but not on day 18 of EAE (Figures 3H–3J) (healthy control vs. EAE + vehicle day 28 *Grp78 p* < 0.01; *Xbp1s p* < 0.0001; and *Atf4 p* < 0.05). This chronic UPR activation was significantly reduced by C-MANF treatment (Figures 3H–3J) (EAE + vehicle vs. EAE + C-MANF day 28 *Grp78 p* < 0.05; *Xbp1s p* < 0.001; and *Atf4 p* < 0.05), supporting the theory that C-MANF promotes recovery in EAE by reducing chronic activation of the UPR. Interestingly, we did not observe an upregulation of the ATF6 pathway of UPR, but treatment with C-MANF still led to a decrease in *Atf6* expression on both days 18 and 28 (Figure 3K) (EAE + vehicle vs. EAE + C-MANF day 18 and day 28, *p* < 0.05). Compared to vehicle-treated EAE mice, C-MANF significantly reduced the mRNA levels of *Gfap*, proinflammatory microglial marker *Cd11b*, and inflammatory cytokine *Tnf* on day 28 (Figures 3L–3N) (EAE + vehicle vs. EAE + C-MANF day 28 *Gfap p* < 0.001; *Cd11b p* < 0.05; and *Tnf p* < 0.05), but not on day 18. The differences in the effects of C-MANF on neuroinflammation observed on day 18 between the IF and qPCR analyses may be due to the specificity of the tissue analyzed; while the IF analysis was performed on lumbar white matter, the area most affected by immune infiltration in EAE, qPCR analysis was performed on RNA isolated from the entire lumbar spinal cord, which may dilute localized white matter effects from treatment with C-MANF.

### Beneficial effects of C-MANF in the CNS of EAE mice are independent from modulation of peripheral immune cells

**Figure 4:**
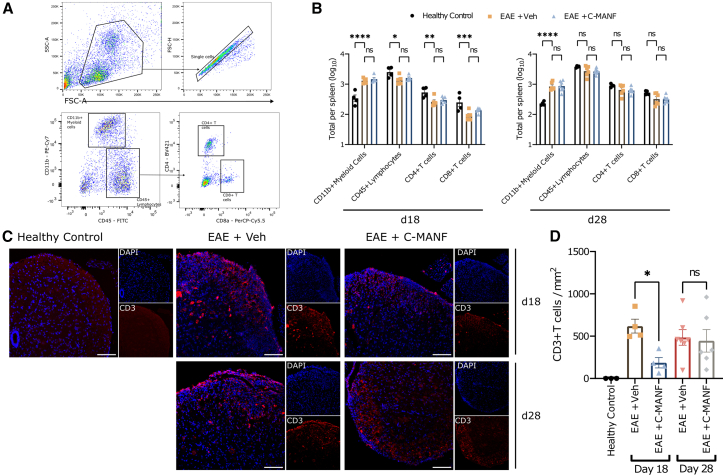
C-MANF does not affect peripheral immune cell populations, but it does limit initial T cell infiltration into the CNS during EAE (A) Gating scheme for flow cytometry analysis of CD11b^+^ myeloid cells, CD45^+^ lymphocytes, CD4^+^ T cells, and CD8^+^ T cell populations. (B) Cellular abundance of gated populations from the spleens of time point-matched controls and EAE mice. (C) Representative IF images stained for CD3 (pan-T cell marker) and DAPI, showing no T cell infiltration in healthy control mice and considerable T cell infiltration in lumbar white matter of EAE mice. Scale bar, 100 μm. (D) Analyzed CD3^+^ T cell counts normalized to analyzed spinal cord area. Bar graphs represent group means ± SEMs; ∗*p* ≤ 0.05; ∗∗*p* ≤ 0.01; ∗∗∗*p* ≤ 0.001; ∗∗∗∗*p* ≤ 0.0001. (B and C) (B) Two-way ANOVA and (C) one-way ANOVA, followed by Holm-Šidak’s post hoc test.

The UPR plays an important role in regulating peripheral immune responses and contributes to chronic autoimmunity.^30^ As EAE is a T cell-driven autoimmune disease model, and T cell differentiation and expansion are known to be affected by UPR activation,^66^^,^^67^ it stands to reason that UPR modulation by C-MANF may affect peripheral immune responses in EAE. To ascertain whether the therapeutic effect of C-MANF administration on EAE mice was caused by peripheral immunosuppression, we used flow cytometry to measure the levels of CD45^+^ leukocytes, CD4^+^/CD8^+^ T cells, and CD11b^+^ myeloid cells in the spleens of EAE mice at the peak (day 18) and chronic (day 28) phases of EAE (Figure 4A). At peak EAE, compared with healthy control mice, vehicle-treated mice exhibited a significant increase in CD11b^+^ myeloid cells (Figure 4B; *p* < 0.0001) and a significant decrease in CD45^+^ lymphocytes (*p* < 0.05), CD4^+^ T cells (*p* < 0.01), and CD8a^+^ T cells (*p* < 0.001) (Figure 4B) (day 18; ANOVA F(3, 33) = 160). By day 28 of EAE, vehicle-treated mice still exhibited a significant increase in CD11b^+^ myeloid cells (*p* < 0.0001), but they did not exhibit significant changes in lymphocyte or CD4^+^/CD8^+^ T cell counts (Figure 4B) (day 28; ANOVA F(1.15, 16.1) = 192). C-MANF-treated EAE mice did not differ from vehicle-treated EAE mice in any cell population count at either time point, indicating that C-MANF had no effect on peripheral immune cell populations (Figure 4B).

Next, we stained EAE spinal cords for CD3, a pan-T cell surface marker, to determine whether C-MANF influenced infiltrating T cell counts. While we could not detect any CD3^+^ T cell infiltration in healthy control spinal cords, CD3^+^ cells were present in the white matter of all EAE spinal cords (Figure 4C). Using automated cell counting, we determined that the level of CD3^+^ T cell infiltration was significantly decreased on day 18 by treatment with C-MANF (Figure 4D) (ANOVA F(4, 19) = 4.284; EAE + vehicle vs. EAE + C-MANF day 18 *p* < 0.05), whereas there was no difference between vehicle-treated and C-MANF-treated mice on day 28. These data suggest that while the administration of C-MANF in EAE mice did not affect peripheral immune cell populations, it could reduce the level of T cell infiltration during the initial peak of the disease course.

### C-MANF enhances remyelination and suppresses neuroinflammation in an organotypic slice model

**Figure 5:**
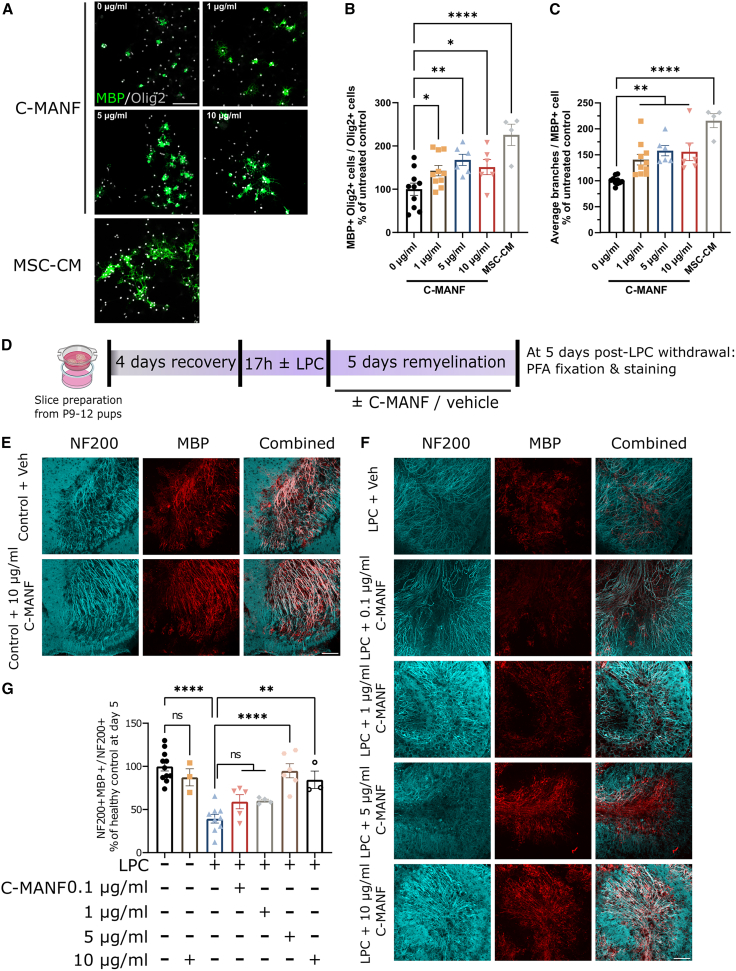
C-MANF enhances OPC differentiation and *ex vivo* remyelination (A) Representative IF images stained for MBP and Olig2, featuring rat primary OPCs cultured for 6 days in differentiation media ± C-MANF at various doses or MSC-CM as positive control. (B) Percentage of Olig2^+^ cells double-positive for MBP, each dot corresponding to an average from three technical replicates. (C) Average number of branches for each MBP^+^ cell, each dot corresponding to an average from three technical replicates. (D) Diagram of timeline for organotypic slice experiments. (E) Representative IF images stained for NF-200 and MBP, featuring naive non-demyelinated slices treated with vehicle or a high dose of C-MANF for 5 days. (F) Representative IF images stained for NF-200 and MBP, featuring slices demyelinated with LPC for 17 h, and then treated with vehicle or C-MANF at various doses for 5 days. (G) Analyzed fraction of myelinated NF-200^+^ axonal fibers in cerebellar slices, each dot corresponding to a single well (average of two slices/well, two images/slice) and reported as the percentage of the average of the naive untreated control group. (A, E, and F) Scale bar, 100 μm. Bar graphs represent group means ± SEMs; ∗*p* ≤ 0.05; ∗∗*p* ≤ 0.01; ∗∗∗*p* ≤ 0.001; ∗∗∗∗*p* ≤ 0.0001. (B, C, and G) One-way ANOVA, followed by Holm-Šidak’s post hoc tests.

OPC differentiation failure is a relevant handicap for remyelination in MS patients. To study the direct effects of C-MANF on CNS remyelination, we first examined whether C-MANF affects OPC differentiation (Figure 5A). We treated primary differentiating rat OPCs^18^^,^^68^ with C-MANF, using mesenchymal stem cell-derived conditioned media (MSC-CM) as a positive control known to enhance OPC differentiation^69^^,^^70^^,^^71^^,^^72^ and compared the fraction and branching morphologies of oligodendroglia expressing MBP. Surprisingly, we found that treatment with C-MANF significantly enhanced the differentiation percentage of OPCs at all tested doses, as did MSC-CM (Figure 5B) (ANOVA F(4, 31) = 7.365; 0 μg/mL vs. 1 μg/mL C-MANF *p* < 0.05; 0 μg/mL vs. 5 μg/mL C-MANF *p* < 0.01; 0 μg/mL vs. 10 μg/mL C-MANF *p* < 0.05; and 0 μg/mL C-MANF vs. MSC-CM *p* < 0.0001). Similarly, treatment with both C-MANF and MSC-CM increased the average number of branches per MBP-expressing cell, with a stronger effect size from MSC-CM (Figure 5C) (ANOVA F(4, 31) = 13.55; 0 μg/mL vs. 1 or 5 or 10 μg/mL C-MANF *p* < 0.01; and 0 μg/mL C-MANF vs. MSC-CM *p* < 0.0001).

To observe whether C-MANF could enhance remyelination following demyelination, we utilized a commonly used *ex vivo* model of lysophosphatidylcholine (LPC)-induced demyelination.^73^ Organotypic cerebellar slices were derived from the brains of postnatal day 9–12 mouse pups and cultured on semiporous membranes. After a 4-day recovery period, the samples were treated with 0.1% LPC for 17 h, followed by the removal of LPC and treatment with either C-MANF or vehicle for 5 days (Figure 5D). Colocalization analysis of MBP and NF-200-immunostained slices showed that treatment with a high dose of C-MANF had no effect on myelination in naive slices (Figure 5E). Treatment with LPC induced a consistent demyelination in the slices compared to that in the naive controls, which was still apparent on day 5 after LPC removal. While low doses (0.1 and 1 μg/mL) of C-MANF did not improve recovery, treatment with 5 or 10 μg/mL C-MANF led to a significant improvement in the ratio of myelinated axonal fibers in the slices (Figure 5F) (ANOVA F(6, 34) = 13.97; LPC + vehicle vs. LPC + 5 μg/mL C-MANF *p* < 0.0001; LPC + vehicle vs. LPC + 10 μg/mL C-MANF *p* < 0.01).

Corresponding with the observed demyelination induced by LPC, staining slices for Olig2 (oligodendroglial lineage marker) and TPPP (mature oligodendrocyte marker) revealed a significant reduction in both total oligodendroglial cells (Figure S3A) (ANOVA F(2, 20) = 8.652, *p* < 0.01), mature oligodendrocyte counts (Figure S3A) (ANOVA F(2, 20) = 31.66, *p* < 0.0001), and the fraction of differentiated oligodendroglia (Figure S3A) (ANOVA F(2, 20) = 3.897, *p* < 0.05) in LPC-treated slices at 24 h after LPC withdrawal. Treatment with C-MANF had no effect on this loss of oligodendrocytes. At day 5, Olig2^+^ cell counts were restored in all LPC-treated slices, and treatment with C-MANF led to a significant increase in TPPP^+^ oligodendrocytes (Figure S3B) (ANOVA F(2, 21) = 6.066, *p* < 0.05) and the fraction of differentiated oligodendroglia (Figure S3B) (ANOVA F(2, 21) = 11.41, *p* < 0.01).

**Figure 6:**
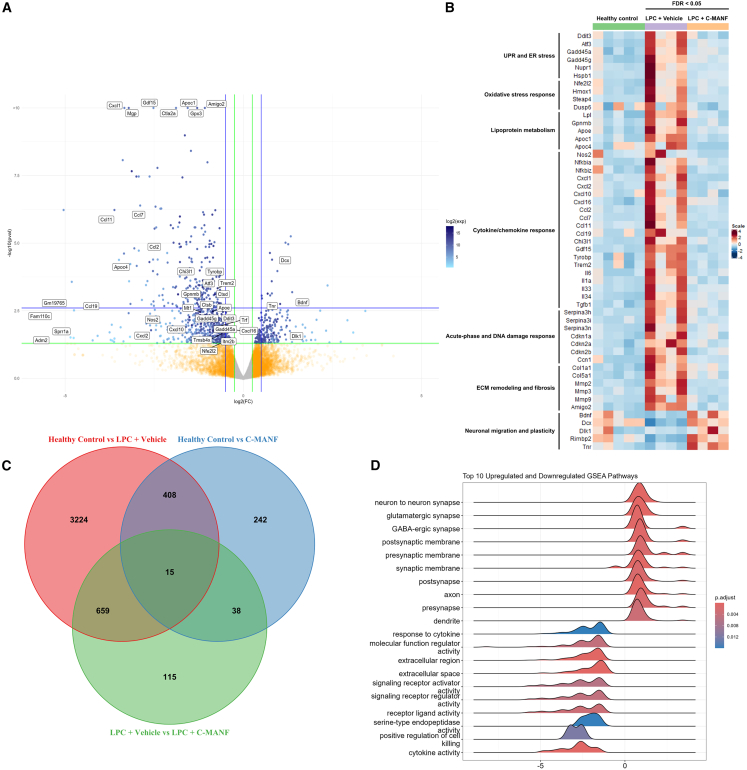
C-MANF shifts the expression of CNS response pathways from proinflammatory toward pro-regenerative during remyelination (A) Volcano plot of −log10 of adjusted *p* values against log2-fold change. Blue dots indicate differentially expressed genes (DEGs) between LPC + C-MANF vs. LPC + vehicle groups at 5 days post-LPC, with selected genes additionally labeled. Green lines indicate threshold boundaries for the adjusted *p* value of 0.05 and fold change of ±0.5, blue lines indicating double of the threshold boundary. (B) Heatmap of the individual sample expression of a selected panel of genes, grouped by their labeled roles and colored by *Z* score normalization. All shown genes are differentially LPC + C-MANF vs. LPC + vehicle groups. (C) Venn diagram showing DEGs co-expressed between comparisons of all treatment groups. (D) Ridgeplot visualizing the 10 most upregulated and downregulated gene set pathways between LPC + C-MANF vs. LPC + vehicle, generated by GSEA. FDR, false discovery rate.

To understand how C-MANF improves remyelination following demyelination by LPC, we performed unbiased mRNA sequencing (mRNA-seq) on RNA collected from cerebellar slices at day 5 following LPC withdrawal. Visualization of differentially expressed genes between vehicle-treated and C-MANF-treated slices shows that treatment with C-MANF led to a widespread downregulation of genes involved in UPR, proinflammatory activation, and fibrosis, while upregulating genes involved in neuronal regeneration (Figures 6A and 6B). Overall, treating slices with LPC led to the differential expression of 4,306 genes vs. healthy controls, 423 (9.8%) of which were also differentially expressed between LPC + C-MANF and healthy controls, and 674 (15.7%) were further differentially expressed between C-MANF and vehicle-treated slices (Figure 6C). By performing gene set enrichment analysis (GSEA) on gene sets expressed differentially between treatment with vehicle and C-MANF, we can similarly see that C-MANF significantly upregulated pathways associated with neuronal synapses and axons, while significantly downregulating pathways associated with proinflammatory extracellular signaling (Figure 6D). Compared to healthy control slices, analysis of the most upregulated Gene Ontology and Kyoto Encyclopedia of Genes and Genomes (KEGG) terms indicated ongoing extracellular remodeling and innate immune cell activation in vehicle-treated slices following demyelination with LPC (Figures S4A and S4B). In contrast, treatment with LPC followed by C-MANF resulted in the upregulation of terms associated with glial differentiation, remyelination, and axonal guidance (Figures S4C and S4D), indicating that C-MANF accelerates the shift from proinflammatory tissue responses toward a tissue microenvironment more conducive to regeneration.

Moreover, while mRNA-encoding key myelin proteins and their promoter *Myrf* were significantly upregulated in both vehicle and C-MANF-treated slices at 5 days following demyelination with LPC (Figure S5A), protein-level expression of MBP was still suppressed in vehicle-treated slices. Analysis of total protein lysates extracted on days 1 and 5 after LPC withdrawal showed that treatment with C-MANF had no effect on MBP protein levels on day 1, but recovered MBP at day 5 (Figures S5B and S5C).

### C-MANF modulates the UPR by increasing acute activation and suppressing chronic activation

**Figure 7:**
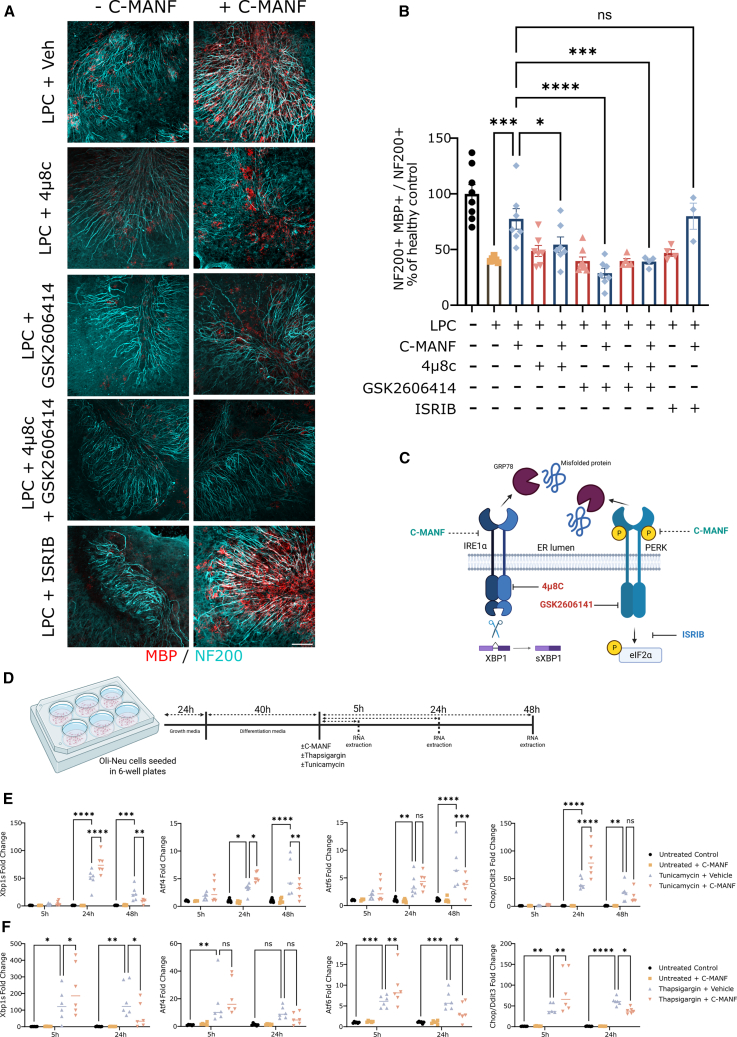
C-MANF induces *ex vivo* remyelination via direct interaction with UPR sensors and reduces chronic UPR activation in oligodendroglia (A) Representative IF images stained for NF-200 and MBP, showing cerebellar slices demyelinated with LPC and allowed to recover with or without 5 μg/mL C-MANF and UPR pathway inhibitors. (B) Analyzed fraction of myelinated NF-200^+^ axonal fibers in cerebellar slices, each dot corresponding to a single well (average of two slices/well, two images/slice) and reported as percentage of the average of the naive untreated control group. (C) Visualization of localized effects of C-MANF and the three UPR inhibitors. While C-MANF interacts with UPR pathway sensors in the lumen of the UPR, 4μ8c and GSK2606414 inactivate the sensors by binding to their cytosolic part (inhibiting efficacy of C-MANF), whereas ISRIB acts downstream of PERK, dephosphorylating p-eIF2α (does not inhibit efficacy of C-MANF). (D) Diagram of the timeline for inducing UPR in differentiating Oli-Neu cells. (E and F) (E) qPCR quantification of mRNA expression of UPR pathways after exposure to tunicamycin or (F) thapsigargin, each dot corresponding to a single well. (A) Scale bar, 100 μm. Bar graphs represent group means ± SEMs; ∗*p* ≤ 0.05; ∗∗*p* ≤ 0.01; ∗∗∗*p* ≤ 0.001; ∗∗∗∗*p* ≤ 0.0001. (B and E) One-way ANOVA, followed by Holm-Šidak’s post hoc test. (C and D) Diagrams created in BioRender (https://BioRender.com/6vxteuu).

Next, we utilized UPR pathway inhibitors to study whether the effect of C-MANF is mediated by the modulation of a specific UPR pathway. We observed in the EAE mouse model that treatment with C-MANF suppressed chronic activation of the IRE1α and PERK pathways; therefore, we tested the effect of inhibiting these pathways on C-MANF-induced remyelination. While 4μ8c inhibits the endonuclease activity of IRE1α^74^ and GSK2606414 is a potent direct inhibitor of PERK,^75^ the integrated stress response inhibitor (ISRIB) inhibits the PERK pathway further downstream by dephosphorylating eIF2α.^76^ Both 4μ8c and GSK2606414 have been observed to inhibit the neuroprotective effects of full-length MANF in mouse primary neuron cultures,^50^ but this has not been previously reported in more complex tissue models. By using a similar experimental timeline as shown in Figure 5, we showed that C-MANF, as previously described, significantly improved remyelination at 5 days after removal of LPC (Figures 7A and 7B) (ANOVA F(10,55) = 13.5; LPC + vehicle vs. C-MANF *p* < 0.001). None of the UPR inhibitors used affected remyelination on day 5 after LPC withdrawal, but when administered in combination with C-MANF, inhibition of either or both of the IRE1α and PERK pathways abrogated the ability of C-MANF to enhance remyelination (Figures 7A and 7B) (C-MANF vs. C-MANF + 4μ8c *p* < 0.05; C-MANF vs. C-MANF + GSK2606414 *p* < 0.0001; and C-MANF vs. C-MANF + 4μ8c + GSK2606414 *p* < 0.001). Inhibition downstream of PERK using ISRIB had no effect on C-MANF-induced remyelination. These data indicate that the effect of C-MANF on the UPR goes beyond simple inhibition and that its neuroprotective effects require the functionality of both the IRE1α and PERK pathway sensors, while downstream modulation of the PERK pathway seemed negligible in this modulation (visualized in Figure 6C).

One of the suggested protective forms of UPR modulation by MANF is the promotion of acute, adaptive UPR activation while inhibiting chronic UPR activation, which is supported by the observation of chronic UPR in MANF-deficient tissues with high protein loads.^41^^,^^77^^,^^78^^,^^79^ As the effects of MANF or C-MANF on acute and chronic UPR in oligodendroglia have not been published before, we tested the effects of simultaneous treatment with C-MANF and the UPR-inducing toxins tunicamycin and thapsigargin in differentiated Oli-Neu cells (Figure 7D) at several time points. Oli-Neu is an immortalized mouse oligodendroglial cell line capable of limited differentiation into myelin-producing oligodendrocyte-like cells.^80^^,^^81^ Tunicamycin had a slower effect on UPR, with no significant changes in UPR detected at 5 h of treatment (Figure 7E). qPCR analysis revealed significant increases in the expression of *Xbp1s* (Figure 7E) (ANOVA F(3, 56) = 54.51; 24 h control vs. tunicamycin + vehicle *p* < 0.0001; 48 h control vs. tunicamycin + vehicle *p* < 0.001), *Atf4* (Figure 7E) (ANOVA F(3, 55) = 22.03; 24 h control vs. tunicamycin + vehicle *p* < 0.05; 48 h control vs. tunicamycin + vehicle *p* < 0.0001), *Atf6* (Figure 7E) (ANOVA F(3, 55) = 19.51; 24 h control vs. tunicamycin + vehicle *p* < 0.01; 48 h control vs. tunicamycin + vehicle *p* < 0.0001), and *Chop (Ddit3*, Figure 7E) (ANOVA F(3, 57) = 39.46; 24 h control vs. tunicamycin + vehicle *p* < 0.0001; 48 h control vs. tunicamycin + vehicle *p* < 0.01) after 24- and 48-h treatment with tunicamycin. As hypothesized, at the acute 24-h time point, C-MANF induced a further significant increase in the expression of *Xbp1s* (tunicamycin + vehicle vs. tunicamycin + C-MANF *p* < 0.0001), *Atf4* (tunicamycin + vehicle vs. tunicamycin + C-MANF *p* < 0.05), and *Chop* (tunicamycin + vehicle vs. tunicamycin + C-MANF *p* < 0.0001), while significantly downregulating *Xbp1s* (tunicamycin + vehicle vs. tunicamycin + C-MANF *p* < 0.01), *Atf4* (tunicamycin + vehicle vs. tunicamycin + C-MANF *p* < 0.01), and *Atf6 (*tunicamycin + vehicle vs. tunicamycin + C-MANF *p* < 0.001), at 48 h. As a faster-acting UPR toxin compared to tunicamycin, treatment with thapsigargin induced the significant upregulation of all observed UPR pathways already at 5 h, with sustained UPR pathway activation at 24 h (Figure 7F). Accordingly, simultaneous treatment with C-MANF increased the expression of *Xbp1s*, *Atf6*, and *Chop* at the acute 5-h time point, while downregulating the expression of *Xbp1s* (Figure 7F) (ANOVA F(3, 40) = 13.71; thapsigargin + vehicle vs. thapsigargin + C-MANF 5 h *p* < 0.05; 24 h *p* < 0.05), *Atf6* (Figure 7F) (ANOVA F(3, 40) = 27.42; thapsigargin + vehicle vs. thapsigargin + C-MANF 5 h *p* < 0.01; 24 h *p* < 0.05), and *Chop* (Figure 7F) (ANOVA F(3, 39) = 34.86; thapsigargin + vehicle vs. thapsigargin + C-MANF 5 h *p* < 0.01; 24 h *p* < 0.05) at the 24-h time point. This finding supports the theory that C-MANF can directly modulate the UPR in oligodendroglia by increasing the acute expression and then suppressing the chronic activation of all three UPR pathways, as well as their proapoptotic downstream target CHOP. C-MANF had no effect on UPR pathway activation in naive cells.

### CNS tissue lacking endogenous MANF is unable to resolve demyelination

**Figure 8:**
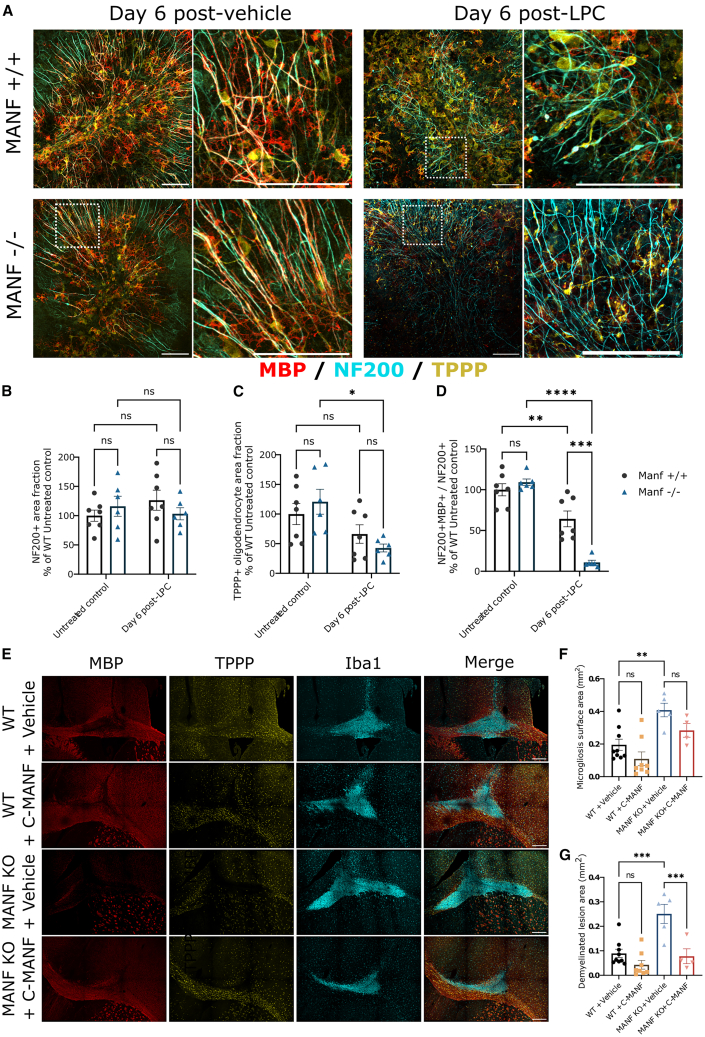
Endogenous MANF is not required for developmental myelination, but it is essential for demyelinating lesion response (A) Representative 20× magnification IF images stained for NF-200, MBP, and TPPP, featuring cerebellar slices from wild-type controls as well as mice featuring full (−/−) knockout (KO) of endogenous MANF, 6 days after treatment with LPC or vehicle for 17 h. Magnified images enlarged from areas highlighted with white dashed boxes. (B–D) Analyzed area fractions of (B) NF-200, (C) TPPP, and (D) myelinated NF-200^+^ axonal fibers in cerebellar slices, each dot corresponding to pooled averages from a single animal (average of four slices/well, one image/slice) and reported as percentage of the average of the *Manf*^+/+^ untreated control group. (E) Representative 2 × 3 tile scan images from the corpus callosum of LPC-injected *Manf*^+/+^ and *Manf*^−/−^ mice, stained for MBP, TPPP, and Iba1, show more extensive demyelination and microgliosis in MANF KO mice. (F and G) Analyzed surface areas of (F) Iba1^+^ microgliosis and (G) demyelination. (A) Scale bars for all images, 100 μm. (E) Scale bar, 250 μm. Bar graphs represent group means ± SEMs; ∗*p* ≤ 0.05; ∗∗*p* ≤ 0.01; ∗∗∗*p* ≤ 0.001. (B–D and F and G) One-way ANOVA, followed by Holm-Šidak’s post hoc test.

Given that tissue lacking endogenous MANF is unable to resolve chronic UPR, we aimed to assess the consequences of demyelination in MANF-deficient tissue. To achieve this, we prepared cerebellar organotypic brain slices from MANF knockout (KO; *Manf*^−/−^) mouse pups.^77^ The slices were genotyped after preparation and maintained for 6 days post-LPC withdrawal. Analysis of whole-mount-stained slices revealed that while *Manf*^−/−^ brain slices exhibited normal axonal morphology and myelination in the absence of LPC, all homozygous KOs were completely altered at 6 days after LPC treatment, as indicated by shrinkage and a total lack of remyelination (Figure 8A). IF analysis revealed that the area fraction of TPPP^+^ oligodendrocytes in *Manf*^−/−^ brain slices remained significantly lower compared to untreated controls (Figure 8B) (TPPP^+^ area fraction (ANOVA F(1, 22) = 11.66; untreated *Manf*^−/−^ vs. LPC-treated *Manf*^−/−^ *p* < 0.05), while the ratio of axonal fibers that were myelinated at 6 days following LPC treatment was significantly suppressed in *Manf*^−/−^ slices compared to WT controls (Figure 8B, NF200^+^MBP^+^/NF200^+^ fraction (ANOVA F(1, 22) = 9.939; LPC-treated *Manf*^*+/+*^ vs. *Manf*^−/−^ *p* < 0.001).

Finally, to demonstrate demyelination-induced degeneration *in vivo*, we induced demyelination in the corpus callosum of *Manf*^−/−^ mice and wild-type (WT) controls using LPC. Analysis of brains collected at day 6 postinjection revealed significantly greater areas of microgliosis (Figure 7E) (ANOVA F(3, 22) = 9.05; *Manf*^+/+^ + vehicle vs. *Manf*^−/−^ + vehicle *p* < 0.01) and demyelination (Figure 7F) (ANOVA F(3, 22) = 13.05; *Manf*^*+/+*^ + vehicle vs. *Manf*^−/−^ + vehicle *p* < 0.001) in MANF-deficient brains. This could be partially rescued by daily s.c. injections of 1 μg/g C-MANF starting on the day after LPC administration, which led to significantly reduced demyelination in *Manf*^−/−^ mice (Figure 7F) (*Manf*^−/−^ + vehicle vs. *Manf*^−/−^ + C-MANF *p* < 0.001).

## Discussion

In this study, we introduce C-MANF, a C-terminal fragment of MANF, as a potential UPR-modulating treatment for MS. We show that s.c.-administered C-MANF enhances regeneration in the CNS of mice suffering from MS-like autoimmune demyelination. Our data showed that C-MANF achieves this by reducing neuroinflammation via UPR modulation in glial cells and increasing the production of remyelinating oligodendrocytes. These positive effects are likely due to direct interactions in the CNS, as (1) we did not observe peripheral immunosuppression as a result of systemic C-MANF administration, and (2) experiments showing enhanced remyelination in *ex vivo* brain slices show that C-MANF is not reliant on peripheral immunomodulation for improving CNS regeneration. Additionally, we showed that C-MANF directly increases primary OPC differentiation and inhibits chronic UPR in an oligodendroglial cell line. Thus, we hypothesize that C-MANF enhances remyelination in EAE mice both by directly supporting OPC differentiation and by removing neuroinflammatory barriers to remyelination. MANF has been shown previously to promote anti-inflammatory pathways via NF-κB modulation,^42^^,^^52^ which may explain the neuroinflammation-dampening effects of C-MANF in EAE. The widespread suppression of proinflammatory pathways in cerebellar organotypic slices revealed by mRNA-seq certainly validates the claim that MANF can modulate the NF-κB inflammasome, yet enhancing remyelination in organotypic brain slices required the functionality of both the IRE1α and the PERK pathways. This finding strengthens our initial hypothesis that the beneficial effects of exogenous C-MANF are entirely UPR mediated, but leaves open the possibility for UPR-independent pro-survival mechanisms in the case of endogenous MANF.

The UPR is a highly evolutionarily conserved cellular response mechanism that is capable of adjusting ER protein folding loads, lipid synthesis rates, and eventually cell death mechanisms as a response to both intracellular and extracellular stimuli. Dysfunction of the UPR in response to the accumulation of misfolded pathological proteins is a hallmark of several neurodegenerative diseases, and in MS it is especially present in glial cells.^12^^,^^34^ Immune cell-derived inflammatory mediators such as interferon-γ induce the UPR, leading to a remyelination-inhibiting differentiation block in oligodendrocytes and OPCs.^82^^,^^83^ Therefore, remyelination could be enhanced by alleviating the UPR in these cells. The most promising current pharmacological approaches to modulating the UPR involve the PERK pathway, particularly prolonging proteostasis-inducing phosphorylation of the initiation factor eIF2α.^19^^,^^84^^,^^85^^,^^86^ Of these, Sephin1, which may function by preventing the dephosphorylation of eIF2α but may also suppress other UPR pathways,^87^ has shown great promise in protecting oligodendrocytes from inflammation-induced apoptosis in animal models of demyelination. C-MANF has a different and seemingly opposite effect on the UPR, reducing the length of UPR pathway activation instead of prolonging it. This difference in mechanism may also explain the difference between C-MANF and Sephin1 when given to EAE mice: prophylactically administered Sephin1 significantly delays EAE onset and initially protects oligodendrocytes from apoptosis but does not reduce peak severity or lead to improved therapeutic outcomes.^19^ The explanation given by Chen et al. for the lack of positive endpoints with Sephin1 is that the occurrence of overwhelming neuroinflammation at later EAE stages causes prolonged eIF2α phosphorylation to become proapoptotic. In a follow-up study, the initiation of Sephin1 treatment at peak EAE resulted in the presence of thinner myelin sheaths in the spinal cord,^85^ often considered an indicator of remyelination, whereas with C-MANF we observed the presence of more and thicker myelin sheaths, which could be a result of a combination of enhancing remyelination and protecting existing sheaths by reducing neuroinflammation. This multifaceted effect may be due to simultaneous modulation of several UPR pathways, rather than just one, as is the case with Sephin1.

Treatment with UPR-activating toxins and C-MANF concurrently increased the expression of genes associated with all three UPR pathways in Oli-Neu cells at early time points, while suppressing the same pathways after prolonged UPR activation. This mechanism of action, capable of downregulating all three UPR pathways during chronic UPR while boosting short-term adaptive UPR, is completely unique to MANF (and possibly CDNF^88^). In fact, excessive chronic UPR is an outcome of MANF ablation in several tissues, including pancreatic islets and midbrain dopaminergic systems.^78^^,^^79^ As shown by Pakarinen et al., loss of MANF in the CNS does not induce neurodegeneration or behavioral changes despite chronic UPR activation, particularly in the IRE1α pathway. However, it potentiates UPR responses to thapsigargin treatment in primary neurons *in vitro*, especially the splicing of *Xpb1* pre-mRNA.^79^ This finding suggests that the chronic UPR itself is not sufficient to induce tissue damage but that it requires an additional trigger. As further evidence of this, we show here that organotypic cerebellar brain slices lacking MANF are unable to remyelinate following demyelination with LPC, and in the corpus callosum of *Manf*^−/−^ mice treatment with LPC leads to extensive demyelination and microgliosis. Thus, we hypothesize that endogenous MANF restricts the timescale of the UPR in response to toxin-induced demyelination, restricting chronic UPR-driven neuroinflammation.

Reducing chronic UPR in astrocytes and microglia in general has been previously shown to suppress autoimmune neuroinflammation.^12^^,^^89^ IRE1α endonuclease activity, especially upregulated in the brains of *Manf*^−/−^ mice as mentioned before, is also upregulated and pathogenic in astrocytes from MS patient samples. A recent analysis of broad rim lesions in MS patients found elevated expression of UPR genes linked to chronic innate immune activation in the myeloid cell rim of this lesion type associated with rapid disease progression.^36^ These findings indicate that chronic UPR pathway activation drives pathogenic astroglial and microglial immune responses that inhibit lesion regeneration. Further single-cell studies are needed to elucidate the precise downstream effects of chronic UPR modulation by C-MANF in diverse glial cell populations at different CNS regions and at different time points. For example, mRNA-seq of LPC-treated slices revealed that C-MANF affected a wide selection of tissue responses, such as those involved in oxidative stress responses and extracellular matrix remodeling, but it is unclear what cell types these changes are primarily mediated in.

MANF and its homolog CDNF have previously shown positive therapeutic effects in rodent models of Parkinson disease, ALS, and stroke^48^^,^^88^^,^^90^^,^^91^ when delivered surgically into the CNS, an administration route that effectively rules out long-term treatment for MS. While not structurally, functionally, or evolutionarily related to MANF, traditional neurotrophic factors such as glial cell line-derived neurotrophic factor have faced challenges in clinical application due to their poor bioavailability and diffusion in tissue, requiring invasive administration when used to treat neurodegeneration in both disease models and human patients. In our previous work, we determined that peripheral administration of MANF results in limited efficacy in treating EAE.^54^ In contrast, reducing the size of MANF to 63 aa from the C-terminal region results in a compound that can be given s.c. and that achieves greatly improved therapeutic outcomes in EAE. At 7.27 kDa, the resulting C-MANF is still too large for conventional passive mechanisms to penetrate the BBB, and it remains unclear how C-MANF enters the CNS. Working with C-CDNF and an even further-truncated version of CDNF, Kulesskaya et al. showed that the influx of the shortened peptide through an *in vitro* BBB model was multiple times greater than the efflux, indicating the presence of active transport mechanisms in the endothelial/astrocyte cell layers of the BBB.^55^ As these peptides are structurally considerably similar to C-MANF, it seems plausible that the same mechanisms allow for influx of C-MANF through the BBB, but this needs to be validated in further studies.

Similarly, the mechanism facilitating the entrance of exogenous C-MANF into the cell compartment is still unclear. A study has shown that binding of human or *Caenorhabditis elegans* MANF to sulfatides in the outer leaflet of the cell membrane facilitates cellular uptake and the cytoprotective properties of MANF.^92^ However, as the N-terminal saposin-like domain of MANF is responsible for this sulfatide binding, this contradicts the improved cytoprotection observed with C-MANF.^58^^,^^92^ It is generally accepted that MANF and CDNF do not affect unstressed cells, and we show here that C-MANF does not modulate UPR pathway activation in naive Oli-Neu cells or affect myelination in healthy organotypic brain slices, and thus any possible cellular transport mechanisms may be tied to increased UPR. C-MANF retains an intact KDEL ER-retention signal at its C terminus, and binding to KDEL receptors is required for the neuroprotection conveyed by both MANF and CDNF.^59^^,^^93^ KDEL receptors are known to traffic proteins between the plasma membrane and the ER via endocytosis, so their binding to exogenous C-MANF may permit the translocation of C-MANF to the ER, where it interacts with UPR sensors.^51^^,^^94^

In summary, C-MANF, a C-terminal fragment of MANF with greatly improved bioavailability *in vivo*, significantly improved clinical recovery in EAE mice via improved remyelination and suppression of neuroinflammation in the CNS. We also showed that endogenous MANF is a key player in restoring tissue homeostasis following demyelination. This is a result of a novel form of UPR modulation, and presents a new therapeutic avenue for treating diseases featuring autoimmune demyelination, such as MS.

## Materials and methods

### Study design

The objective of this study was to examine the neurorestorative potential of C-MANF, a C-terminal fragment of MANF, in various *in vivo*, *ex vivo*, and *in vitro* models of MS. We used the EAE mouse model to examine the effects of C-MANF on experimental autoimmunity-driven demyelination, by analyzing (1) clinical score, body weight gain, and motor behavior; (2) ultrastructural TEM analysis of myelination; (3) IF-stained spinal cords using antibody markers for myelin, axons, glial cells, infiltrating T cells and chronic UPR; and (4) RNA and proteins extracted from spinal cords using qPCR and western blot. Primary OPCs from rat pups were used to evaluate the drug-induced effects on OPC differentiation. Organotypic cerebellar brain slices were cultured and demyelinated with LPC to examine drug-induced effects on remyelination. Oli-Neu cells were used to study the timescale of the effect of C-MANF on UPR pathway activation in oligodendroglia using 3′ mRNA-seq. Finally, we used *Manf*^−/−^ mice to observe the outcome of demyelination without the presence of endogenous MANF in organotypic slices or in the corpus callosum.

Animal experiments were performed according to the 3R principles of the European Union Directive 2010/63/EU regarding the care and use of experimental animals and following local laws and regulations (Finnish Act on the Protection of Animals Used for Scientific or Educational Purposes [497/2013], Government Degree on the Protection of Animals Used for Scientific or Educational Purposes [564/2013]). The experimental protocols were authorized by the National Animal Experiment Board of Finland (license nos. ESAVI/11997/2023 and ESAVI/27113/2020). Animals were housed under a 12:12 light/dark cycle, and water and food were available *ad libitum*. EAE experiments were performed in two sets, the sample sizes of which were determined from previous experiments and are indicated in the figure legends. Animal experiments were reported in accordance with the ARRIVE (Animal Research: Reporting of *In Vivo* Experiments) guidelines.

All the specific methods and details of the models and protocols used are described in the supplemental information.

### Statistical analysis

Statistics for all data were analyzed using GraphPad Prism (version 9.2, GraphPad Software, USA). The statistical methods we used are described in the figure legends. The data were generally analyzed with two-way or one-way ANOVA, followed by Dunnett’s test for clinical analyses, Holm-Šidak’s test for IF, western blot, qPCR, and flow cytometry analyses. The Kruskal-Wallis test, followed by Dunn’s test, were used for nonparametric IF data, two-tailed unpaired *t* tests were used when comparing only two groups together, and REML estimation was used for matched clinical results with missing data points. Data were reported as the percentage of healthy/untreated control population mean when denoted on graph *y* axis. Data were usually represented as group means ± SEMs, with individual data points shown in graphs whenever possible. Results were considered significant when *p* ≤ 0.05.

### Reagents

Chemicals were acquired from Sigma-Aldrich (USA) unless otherwise specified. C-MANF was ordered as a 63-aa chemically synthesized peptide from Apeptide (China), with the aa sequence of KYDKQIDLSTVDLKKLRVKELKKILDDWGETCKGCAEKSDYIRKINELMPKYAPKAASARTDL. C-MANF was stored at −80°C and diluted in PBS for experiments.

### EAE animal model

#### EAE induction

Female 6-week-old C57BL/6JRccHsd mice from Envigo were habituated to animal facilities for 2 weeks prior to EAE induction. EAE was induced using the Hooke Laboratories EK-2110 EAE induction kit, according to the manufacturer’s instructions. In addition to two s.c. injections of myelin oligodendrocyte glycoprotein (MOG)_35–55_/complete Freund’s adjuvant (CFA) emulsion, mice received 120 ng pertussis toxin intraperitoneally (i.p.) at 2- and 24-h time points. For healthy control animals, MOG/CFA was replaced by an equal volume of PBS.

#### Scoring and treatment

Mice were weighed and scored daily according to Hooke Laboratories’ EAE scoring guide, in which 0 = no symptoms, 1 = limp tail, 2 = weak hindlimbs, 3 = completely paralyzed hindlimbs, 4 = partial front leg paralysis, and 5 = mouse found dead due to paralysis (scores between integers [e.g., 2.5] indicate a clinical picture between the two numbered scores). Individual mice were rolled into blinded treatment groups balanced by score and body weight upon showing EAE symptoms corresponding to a clinical score of 1; mice which failed to show symptoms of EAE were excluded (two mice in each set) by the end of the experiment. Mice in EAE treatment groups received 1 μg/g C-MANF (i.e., a 20-g mouse received 20 μg of C-MANF in a single injection), 4 μg/g C-MANF (only in the first set of EAE experiments), or vehicle (PBS) as daily s.c. bolus injections of 5 μL/g. Researchers performing animal experiments were blinded to EAE treatment groups but not the healthy control group. Doses for pilot experiments were selected based on earlier experiments using hMANF^54^ and adapted for s.c. use by normalizing to mouse body weight.

### Analysis of motor function

Behavioral testing was carried out at the Mouse Behavioral Phenotyping Facility, supported by Biocenter Finland and HiLIFE. Rotarod (Ugo Basile) and open field (ENV-520, Med Associates Inc.) tests were performed to evaluate motor function deficit.

#### Rotarod

Mice were trained once per day for 2 days prior to EAE nduction on an accelerating rotating platform (from 4 to 40 rpm for a maximum of 240 s). Once a mouse fell off the rotating rod, the latency to fall (time it takes for the mouse to drop from the rod) was recorded. During the experiment, mice were tested at days 14, 21, and 28 after induction.

#### Open field

Spontaneous locomotor activity was tested at the chronic EAE time point of day 28. Mice were placed in an enclosed, lit arena, and their ambulation speed, time, and counts, as well as vertical rearing counts were recorded for 10 min.

### Euthanasia and tissue processing

At the endpoint (18 or 28 days) of the EAE experiments, all mice were sedated with terminal anesthesia using sodium pentobarbital (90 mg/kg, i.p.; Orion Pharma, Finland).

#### TEM sample preparation

EAE mice were perfusion fixed with 2% glutaraldehyde (electron microscopy [EM] grade) and 2% formaldehyde (EM grade) in 0.1 M sodium phosphate buffer, pH 7.4. Lumbar spinal cords were dissected and cut into approximately 200-μm vibratome sections in ice-cold phosphate buffer. Tissue sections were further fixed with 2% glutaraldehyde and 2% formaldehyde in phosphate buffer for 4 h in a refrigerator. Prior embedding, samples were stored in 2% formaldehyde in phosphate buffer in a refrigerator over a weekend. Tissue sections were then washed with phosphate buffer, prior to osmication with 1% reduced osmium tetroxide in 0.1 M phosphate buffer for 2 h on ice. Phosphate buffer was exchanged for distilled water prior to uranyl acetate en bloc staining with 2% aqueous solution in a refrigerator for 1 h, followed by washing with distilled water; gradual dehydration with 20%, 30%, 50%, 70%, 90%, 96%, and 100% ethanol (two times, each step for 8 min); two incubations in acetone (each for 8 min); and gradual infiltration into hard epoxy (TAAB Laboratories, UK) over 2 days and polymerization at 60°C for 1 day.

Semithick sections were cut and stained with toluidine blue for light microscopy to guide the trimming of the target area, where ultrathin 60-nm sections were cut using the Ultracut UCT7 ultramicrotome (Leica Mikrosysteme GmbH, Germany), picked on Pioloform-coated single-slot copper grids, and poststained with uranyl acetate (SPI Supplies, USA) and lead citrate (Leica Ultrostain 2).

#### RNA and protein extraction

Mice were transcardially perfused with cold PBS for 5 min, until the liver was clear of blood. The spine was resected on ice and immediately snap-frozen in dry ice and stored at −80°C. RNA and protein purification was performed using a NucleoSpin RNA/Protein Purification Kit (Macherey-Nagel, USA) following the manufacturer’s recommended protocol. The lumbar spine L2–L6 was resected from the spinal column of the frozen samples and immediately placed into complete lysis buffer supplemented with 2-mercaptoethanol. The tissue was homogenized by passing through a G20 needle five times, then passing through a G25 needle five times, before finally being centrifuged in the manufacturer-provided filtration column. Following the manufacturer’s protocol, eluted RNA was screened for purity and concentration by nanodrop, and protein samples were assayed using a protein quantification assay (Macherey-Nagel) before being stored at −80°C.

#### IF

EAE mice were transcardially perfused with cold PBS for 5 min, followed by resection of the lumbar spine into cold 4% paraformaldehyde (PFA). After postfixation at 4°C over a weekend, the lumbar spinal cord L2–L6 was resected and processed for paraffin embedding with a Leica ASP300S. We sectioned 16-μm slices with a microtome and transferred onto glass slides for staining. LPC-injected mice were transcardially perfused with cold PBS for 5 min, then with cold 4% PFA for another 5 min, after which brains were collected. After postfixation at 4°C over a weekend, brains were processed for paraffin embedding with a Leica ASP300S. We sectioned 10-μm slices with a microtome and transferred them onto glass slides for staining.

#### Flow cytometry

Spleens were harvested from mice under terminal anesthesia prior to perfusion and placed in cold Hank’s balanced salt solution (HBSS). To produce single-cell suspensions, spleens were pushed through 70-μm cell strainers (VWR, USA), which were then washed with 5 mL PBS. Suspensions were centrifuged at 650 × *g* for 5 min, and then cell pellets were resuspended in 500 μL red blood cell (RBC) lysis buffer (BD Biosciences, USA) and incubated for 10 min at room temperature (RT). Suspensions were washed by adding 500 μL PBS and centrifuging at 650 × *g* for 5 min twice, after which they were resuspended in 100 μL cell staining buffer (BD Biosciences).

### Primary OPC and MSC cultures

#### Primary OPC culture

OPCs were obtained from postnatal day (P) 3-6 Sprague-Dawley rats of both sexes. Pups were decapitated and brains removed quickly and kept in ice-cold Hibernate A (Thermo Fisher Scientific, USA). Olfactory bulbs and cerebella were discarded, and brain tissue was mechanically minced using a scalpel. Tissue was then enzymatically digested in dissociation solution (Hibernate A, 15 U/mL papain [Sigma], 20 μg/mL DNAse I [Merck, USA]). Single cells were collected by triturating the tissue suspension using a 5-mL serological pipette and collecting the supernatant, which was passed through a 70-μm cell strainer (Falcon, Corning, USA) into a 90% isotonic Percoll (GE Healthcare, USA) solution, which was diluted to 22.5% with neutralizing solution (Hibernate A, 2% B27 [Gibco, USA] and 2 mM sodium pyruvate [Gibco]). Single cells were separated from debris by gradient density centrifugation (800 × *g*, 20 min, RT, without breaks), and myelin debris was discarded. Cell pellet was depleted of erythrocytes by resuspending it in RBC lysis buffer (Roche, USA). Single cells were then labeled with mouse-anti-rat A2B5-IgM (immunoglobulin M) antibody (Millipore, USA) for 30 min at 4°C, rat-anti-mouse-IgM microbeads (Miltenyi Biotec, Germany) for 15 min at 4°C and then sorted through a mass spectrometry column (Miltenyi Biotec) using a MiniMACS Separator (Miltenyi Biotec). Isolated OPCs were seeded at a density of 10,000 cells/well onto 13-mm glass coverslips (VWR) coated with poly-d-lysine (Sigma), and incubated at 37°C, 5% CO_2_ in OPC media composed by Dulbecco’s modified Eagle’s medium (DMEM) F12 (Gibco) plus SATO supplement (60 μg/mL *N*-acetyl cysteine, Sigma), human recombinant insulin (10 μg/mL, Gibco), sodium pyruvate (1 mM, Gibco), apo-transferrin (50 μg/mL, Sigma), putrescine (16.1 μg/mL, Sigma), sodium selenite (40 ng/mL, Sigma), progesterone (60 ng/mL, Sigma), and bovine serum albumin (BSA; 330 μg/mL, Sigma). To promote OPC expansion, media was supplemented with basic fibroblast growth factor (bFGF) and platelet-derived growth factor PDGF (30 ng/mL each, PeproTech, USA) for 48 h. Thereafter, to promote differentiation, OPCs were incubated in OPC media (without bFGF and PDGF), supplemented with thyroid hormone (3,3′,5-triiodo-l-thyronine [T3], 40 ng/mL, Sigma), and various concentrations of C-MANF (0, 1, 5, and 10 μg/mL). During differentiation, 80% of the media was refreshed every 72 h. After 6 days under differentiation conditions, OPCs were fixed with 4% PFA for 10 min at RT. Data were obtained from three independent experiments, with each condition performed in three technical replicates.

#### Primary MSC cultures

MSCs were prepared adapting the protocol from Rivera et al.^70^ Briefly, bone marrow plugs were harvested from the femur and tibias of 2- to 3-month-old Sprague-Dawley rats (Charles River, USA). Plugs were mechanically dissociated in DMEM (Gibco) and recovered by centrifugation, washed with DMEM, and resuspended in proliferation medium (DMEM containing 10% heat-inactivated fetal bovine serum [FBS; Gibco], 1% GlutaMAX [Gibco], and 1% antibiotic-antimycotic [Gibco]). Cells were seeded and cultured in a Petri dish in a humidified incubator at 37°C with 5% CO_2_, and media was changed every 3 days. When seeded cells reached 80% confluence, the proliferation media was removed, cells were washed with 1× PBS (Gibco), and media was replaced with OPC media. After 3 days, MSC-CM was collected and filtered using a 0.22-μm pore filter. MSC-CM was obtained from passages 3–6 and used as media for the positive control group OPCs.

### Organotypic cerebellar brain slice model

To make organotypic cerebellar brain slices, P9–P12 C57BL/6JRccHsd pups of both sexes were euthanized by CO_2_ inhalation, and dissected cerebella were cut into 350-μm slices using a McIlwain Tissue Chopper. Samples were placed on Millicell cell culture inserts (30 mm, 0.4 μm pores) in 6-well plates with serum-supplemented media (SSM) consisting of 50% MEM, 25% HBSS, and 25% horse serum, supplemented with 1% antibiotic-antimycotic, 0.5% l-glutamine, and 0.5% glucose (all SSM reagents from Gibco). Slices from each pup were divided equally into wells, with the number of slices/well in separate experiments indicated in the figure legends. Media was replaced in each well 24 h after preparation and every 48 h subsequently. Demyelination was induced on the 5^th^ day after slice preparation by replacing media with SSM containing LPC (from *Glycine max*) at 0.5 mg/mL for 17 h. LPC was diluted in HBSS and sonicated for 30 min before adding to media. After toxin treatment, media was replaced with SSM, with possible additions of C-MANF or UPR modulators. The 4μ8c (20 μM), GSK2606414 (5 μM) (both from MedChemExpress, USA) and ISRIB (5 μM) (Sigma) were dissolved in DMSO and diluted by 1:500 in SSM for use. SSM for C-MANF and vehicle groups contained a corresponding dilution of DMSO.

After 5 or 6 days of remyelination, slices were fixed for IF analysis of myelination with 4% PFA for 1 h at RT, and then washed with PBS before IF staining. RNA and protein purification was performed using a NucleoSpin RNA/Protein Purification Kit (Macherey-Nagel) following the manufacturer’s recommended protocol, as described earlier.

### Oli-Neu cell line model

Oli-Neu cells (a gift from Prof. Iiris Hovatta’s lab, with permission from Prof. Jacqueline Trotter) were maintained in a simple growth media containing DMEM, 10% FBS, 2 mM l-glutamine, 1× penicillin-streptomycin, 0.1% sodium pyruvate, and 15 mM HEPES (all growth media reagents from Gibco). For analysis of UPR activation in differentiating Oli-Neu, cells were plated in 6-well plates at 250,000 cells/well in a modified DMEM-SATO base growth medium containing DMEM, BSA (0.1 mg/mL, Sigma), putrescine (0.016 mg/mL, Sigma), progesterone (60 ng/mL, Sigma), l-glutamine (2 mM, Gibco), 1× penicillin-streptomycin (Gibco), sodium pyruvate (1 mM, Gibco), 1× insulin-transferrin-selenium (Gibco), *N*-acetyl-l-cysteine (5 μg/mL, Sigma), 1× B-27 (Gibco), 1× Trace Elements B (Corning), d-biotin (10 ng/mL, Sigma), PDGF (20 ng/mL, PeproTech), and NT-3 (1 ng/mL, PeproTech). After 24 h of OPC-like proliferation in 6-well plates, growth media was replaced by DMEM-SATO differentiation media, which had the following modifications from the DMEM-SATO growth media: B-27 concentration lowered to 0.1× and PDGF and NT-3 replaced with PD 174,265 (1 nM, epidermal growth factor receptor tyrosine kinase inhibitor from Cayman Chemicals) and T3 (10 ng/mL, Sigma). After 48 h in differentiation media, toxin-treated groups were supplemented with either 50 nM thapsigargin or 100 nM tunicamycin, and drug-treated groups with 5 μg/mL C-MANF. Total RNA was isolated from differentiated Oli-Neu cells at 5, 24, or 48 h after the beginning of treatment with toxins, C-MANF, and/or corresponding vehicle controls using the TRIzol reagent solution (Thermo Fisher Scientific).

### TEM image acquisition and image analysis

TEM micrographs were acquired using the Hitachi HT7800 microscope (Hitachi High-Technologies, Japan) operated at 100 kV and equipped with a Rio9 CMOS camera (AMETEK Gatan, USA). Micrographs were taken at 2,000× magnification from five randomly selected areas in the ventral white matter of the sections. Myelinated axons were analyzed using the G-ratio plugin for ImageJ software. To summarize, the plugin selects random areas in a micrograph, which when located in an axon leads to the researcher drawing the outline of both the axon and the myelin sheath. Approximately 60–90 myelinated axons were quantified per micrograph, and the software calculated G-ratios as the ratio of axon perimeter divided by myelin sheath perimeter. Myelin thickness was calculated for each sheath by subtracting the axonal inner diameter from the diameter of the full myelin sheath, with an assumption of circularity.

### IF

#### Mounted section staining

Glass-mounted spinal cord or brain sections were deparaffinated using a series of xylene and ethanol, after which antigen retrieval was performed by heating slides in a sodium citrate buffer (10 mM, pH 6.0, 0.05% Tween 20). Sections were washed in Tris-buffered saline (TBS), permeabilized in two changes of TBS-T (TBS with 0.1% Tween 20), and then blocked with 5% BSA and 1% horse serum in TBS-T for 1 h. Sections were then incubated with primary antibodies diluted in TBS-T with 5% BSA overnight at 4°C (see Table S1 for antibodies and concentrations used). After primary antibody incubation, sections were washed three times with TBS-T and then incubated with secondary antibodies diluted in TBS-T with 5% BSA for 1 h at RT. Afterward, sections were possibly incubated with DAPI diluted in TBS, washed in TBS three times, and mounted with coverslips using Immu-Mount (Epredia, USA).

#### Primary OPC staining

Fixed cells were blocked in PBS containing 1% BSA, 0.4% fish skin gelatin buffer, and 0.1% Triton X-100 for 1 h at RT. Primary antibodies were diluted in blocking solution and incubation was performed overnight at 4°C. After washing, cells were incubated in blocking solution containing fluorochrome-conjugated species-specific secondary antibodies (see table below for antibodies and concentrations used) at RT for 2 h and washed three times with PBS. Specimens were mounted on Superfrost Plus (VWR) slides using ProLong Gold antifade reagent (Invitrogen, USA).

#### Whole-mount staining

Fixed organotypic brain slices were cut out of inserts with a scalpel and transferred to 24-well plates with blocking solution containing 0.5% Triton X-100, 5% horse serum, and 1% BSA in PBS for 1 h. Blocking solution was then replaced with primary antibodies diluted in more blocking buffer, and samples were incubated with primary antibodies overnight at 4°C. Samples were then washed three times with blocking solution and incubated with secondary antibodies diluted in blocking solution for 2 h at RT. Secondary antibody controls are visualized in Figure S6. Following a single wash with PBS, samples were incubated with DAPI diluted in PBS and then washed three times with PBS. Finally, samples were transferred onto glass histology slides and gently mounted with coverslips using Immu-Mount (Epredia).

#### Imaging

Laser scanning confocal micrographs of the EAE and organotypic slice samples were acquired with an SP8 Stellaris 8 FALCON (Leica) confocal microscope using an HC PL APO 20×/0.75 CS2 air objective. For higher-magnification analysis of CHOP colocalization with glial cell markers in spinal cord samples, an HC PL APO 40×/1.25 motCORR glycerol objective was used. z stacks of 10 images at 1-μm intervals (2 μm for organotypic brain slices) were taken from the stained area and combined into a maximum intensity projection using the imaging software Las X (version 4.4.0, Leica). Images from fluorescently labeled OPCs were captured using a Zeiss LSM980 confocal microscope. Data were obtained from 4–8 fields per technical replicate.

#### Image analysis

All confocal micrographs were analyzed using Fiji ImageJ software (version 2.14.0/1.54f). For analysis of EAE spinal cords, three z stack images were taken from sequential spinal cord sections at fixed locations in the ventral area of the spinal cord. For the quantification of MBP, NF-200, Iba1, and GFAP area fractions from 20× magnification micrographs, the fraction of positively stained pixels within a region of interest (ventral white matter) was calculated, the results averaged for each spinal cord, and the final values represented as a percentage of the mean of the healthy control group. For counting TPPP^+^ and CD3^+^ cells, Fiji automated cell counting was used to count particles over the size of 10 μm^2^ in the ventral white matter. For the quantification of Iba1^+^CHOP^+^ and GFAP^+^CHOP^+^ area fractions in 40× magnification micrographs, object-based overlap analysis was performed: the Fiji image calculator was used to create an image containing only pixels positive for both Iba1 and CHOP, from which the area fraction in the ventral white matter was calculated. The number of CHOP^+^ oligodendrocytes was counted manually from 40× magnification micrographs as the number of TPPP^+^ cells that were clearly positive for CHOP. The quantification of MBP^+^ oligodendrocytes was performed using an automated Fiji macro. Briefly, confocal images were processed to create maximum intensity projections and separated into channels for DAPI, Olig2, CNPase, and MBP. A threshold for each marker, determined from the most differentiated condition, was applied uniformly across all images from the same experiment. Masks were created for DAPI, Olig2, and CNPase cells, and the Image Calculator function was used to generate composite masks for DAPI^+^/Olig2^+^, called Olig2^+^ cells, and DAPI^+^/Olig2^+^/CNPase^+^ cells. The Fill Holes function was used to fill any voids left by nuclei. A final mask combined both DAPI^+^/Olig2^+^/CNPase^+^ and DAPI^+^/Olig2^+^/MBP^+^ masks, called MBP^+^ cells, to minimize the possibility of MBP^+^ branches overlapping Olig2^+^/CNPase nuclei being counted as mature oligodendrocytes. The counts of MBP^+^ and Olig2^+^ cells were recorded for each image, and averages were calculated for each experimental condition. For the analysis of differentiated OPC branching, MBP-channel images were binarized, skeletonized, and then further analyzed using Fiji. Skeletons with fewer than three branches were excluded, and the number of branches per skeleton was averaged for each technical replicate.

### RNA analysis with RT-qPCR

#### cDNA synthesis

Synthesis of cDNA for qPCR was performed with the previously purified RNA using the commercially available Maxima H Minus First Strand cDNA Synthesis Kit (Thermo Fisher Scientific) following the manufacturer’s protocol. The samples were synthesized using the same concentration of RNA template, resulting in a 50-ng/μL final concentration (for RNA isolated from organotypic brain slices, due to smaller output, a final concentration of 12.5 ng/μL was used for cDNA synthesis).

#### RT-qPCR

Prepared cDNA samples were diluted 1:10 in sterile water and combined with LightCycler 480 SYBR Green I Master Mix (Roche) and primers (see sequences in Table S2) in 384-well plates (10 μL total volume/well). Plates were centrifuged briefly and placed in a LightCycler 480 System (Roche) for RT-qPCR. Results were acquired using the Basic Relative Quantification function in the LightCycler 480 Real-Time PCR System software and analyzed using the ΔΔCt method. All samples were performed in triplicate. Outlier analysis (Grubb’s test at an alpha of 0.05) was performed on all RT-qPCR results, and outliers with an RNA purity (260/230 or 260/280 as measured by nanodrop) below 1.8 were excluded from the final analysis.

### mRNA transcriptomics

#### Sample prep and sequencing

We performed 3′ mRNA-seq using RNA extracted from cerebellar organotypic slices as described earlier. All samples were purified using a NucleoSpin RNA Clean-up Kit (Macherey-Nagel), and RNA integrity evaluated using a TapeStation 4200 (Agilent, USA). Samples selected for sequencing had an RNA integrity number of >8 and were sent to Biomedicum Functional Genomics Unit at the Helsinki Institute of Life Science for sequencing. Library preparation was done using a NEBNext Ultra II Directional RNA Library Prep kit (New England Biolabs, USA) according to the manufacturer’s instructions and sequenced using an Illumina NextSeq 500, generating single end reads of 75 bp for each sample. Reads were aligned onto the reference genome based on GENCODE Mouse Release M36 (GRCm39).

#### Differential expression and pathway analysis

Prior to analysis, genes with <10 total counts across all samples were excluded. Gene counts were analyzed and results visualized with BulkAnalyseR,^95^ using edgeR to examine differential expression of individual genes between treatment groups.^96^ Genes were considered differentially expressed with an adjusted *p* < 0.05 and a fold change of >0.5. Further pathway analysis was performed using clusterProfiler,^97^ allowing for overrepresentation analysis of Gene Ontology and KEGG terms, as well as further GSEA on the most upregulated gene ontologies using fgsea.^98^ For all pathway analyses, the *p* value cutoff was held at 0.05, and *p* values were adjusted for false discovery rate using the Benjamini-Hochberg method. Functional enrichment results were visualized using the dotplot and ridgeplot features in enrichplot.^99^

### Protein analysis with western blot

Equal amounts of protein (20 μg from EAE spinal cords, 10 μg from organotypic brain slices) were separated on 4%–20% Mini-PROTEAN TGX Stain-Free gels (Bio-Rad, USA). After electrophoresis, gels were activated with UV light in a ChemiDoc MP imaging system (Bio-Rad) and protein transferred to 0.2 μm polyvinylidene fluoride (PVDF) using a Transblot Turbo (Bio-Rad). Total protein images were taken from PVDF membranes for normalization using the ChemiDoc MP, after which the membranes were blocked in TBS with 5% BSA for 1 h at RT. Primary incubation with antibodies diluted in PBS-T with 5% BSA was performed at 4°C overnight, Then, membranes were washed with TBS-T and incubated with horseradish peroxidase-conjugated secondary antibodies for 1 h at RT. Stained proteins were visualized using Pierce ECL western blotting substrate (Thermo Fisher Scientific) on the ChemiDoc MP. Semiquantitative analysis of protein quantity relative to total protein stain was performed using Fiji ImageJ, and the results were expressed as the percentage of mean results of healthy control groups.

### Flow cytometry analysis of spleen immune cell populations

#### Flow cytometry staining

Following cell counting, 1 × 10^6^ live cells were collected for each spleen in 100 μL cell staining buffer. Fc receptor-mediated binding was blocked by incubation with Mouse BD Fc Block (1:200, BD Biosciences) for 15 min, after which samples were stained with FITC CD45, BV421 CD4, PerCP-Cy 5.5 CD8a, and PE-Cy7 Anti-Mouse CD11b (BD Biosciences; further antibody information in Table S1) for 30 min at 4°C. Both single fluorophore and FMO (fluorescence minus one) controls were also made for each conjugated antibody, as well as unstained controls, using healthy control animal splenocytes. Following antibody staining, samples were washed by adding 500 μL cell staining buffer and centrifuging at 650 × *g* for 5 min twice. Cells were kept resuspended in 500 μL cell staining buffer overnight, before flow cytometry.

#### Flow cytometry

A BD LSRFortessa Analyzer with the standard four-laser setup was used to analyze samples on the morning following staining. Single fluorochrome, FMO, and unstained controls were used to determine fluorochrome spread into channels and to determine accurate gating for each population of interest (Figure 4A) using BD FACSDiva software (version 8.0). Afterward, populations were analyzed and visualized using FlowJo software (version 10.0, FlowJo, USA).

### Generation of MANF KO mice and genotyping

#### Manf-targeted embryonic stem cells and KO mice

The targeted mouse embryonic stem cell (ESC) clone MANF_D06 (EPD0162_3_D06, C57Bl/6N-Manf tm1a(KOMP)Wtsi) used for generating *Manf*^±^ mice was generated by the trans-NIH Knockout Mouse Project (KOMP) and obtained from the KOMP Repository (www.komp.org). Chimeric mice were obtained from targeted ESCs aggregated with morula stage pre-implantation embryos of the outbred ICR, Swiss CD-1 strain (Envigo) at the Genetically Modified Rodents Unit of the Laboratory Animal Centre, University of Helsinki. Chimeric mice with germline transmission of the targeted MANF construct were crossed with WT ICR mice to generate heterozygous *Manf*^±^ mice. *Manf*^−/−^ and *Manf*^+/+^ mice for experiments were obtained from heterozygous breeding.

#### Genomic DNA isolation and genotyping

For genotyping of animals from earmarks, AccuStart II PCR Genotyping Kit (QuantaBio, USA) was used. Genotyping was carried out using the following PCR primers: MANF KO primers f2 5′-TGG AGTGAG CACAAC TCA GG-3′, r2 5′-GGC TTC GAC ACC TCA TTG AT-3′, and r4 5′-CCA CAA CGG GTT CTT CTG TT-3' (WT f2/r2 PCR product, 547 bp; KO f2/r4 product, 352 bp).

### LPC-induced focal demyelination in mice

As *Manf*^−/−^ mice begin to develop severe diabetes from the age of 7 weeks, 5-week-old male and female *Manf*^−/−^ and *Manf*^*+/+*^ were injected with a volume of 1 μL 1% LPC through unilateral stereotaxic injection in the lateral corpus callosum (coordinates relative to bregma and dura: anteroposterior axis −0.1 mm; lateromedial axis −1 mm; dorsoventral axis −1.6 mm) under isoflurane anesthesia. The injection flow rate was set at a rate of 0.25 μL/min. The needle was slowly removed after a delay of 5 min to avoid backflow of injected LPC. A set of both +/+ and −/− mice were given daily s.c. injections of 1 μg/g C-MANF, starting on the day after LPC injection. Mice were euthanized at 6 days postsurgery, and their brains were collected for IF staining.

## Data and code availability

Data associated with this study are present in the paper or the supplemental information. Further data are available from the corresponding authors upon reasonable request.

## Acknowledgments

We wish to thank Katariina Alasentie, Konsta Valkonen, Janika Sirjala, and Dr. Maria Colpo for their technical help. We would like to thank Dr. Alerie Guzmán de la Fuente for sharing the Fiji macro used for quantitative analysis of the OPC differentiation *in vitro* studies, and Saila Medina Leskinen for data acquisition. We thank the Electron Microscopy Unit of the Institute of Biotechnology, University of Helsinki, for providing sample preparation and access to the microscope. We gratefully acknowledge the HiLife Flow Cytometry Unit for assistance with flow cytometry, access to facilities and expertise at the HiLife BI Histology core facility, as well as excellent support from the HiLIFE Laboratory Animal Center Core Facility, all at the University of Helsinki. mRNA-seq was provided by the Biomedicum Functional Genomics Unit at the Helsinki Institute of Life Science and Biocenter Finland at the University of Helsinki. C.R.R. is part of the Medical Sciences Doctoral Program, Graduate School Faculty of Medicine, Universidad Austral de Chile, Valdivia, Chile. We acknowledge the NIH KOMP for the MANF-targeted ESC clone used to develop the MANF KO mice. This work was supported by grants from the 10.13039/501100000781European Research Council (grant nos. 805426 and 101158120) to M.H.V.; the 10.13039/501100002341Research Council of Finland (grant nos. 309708, 314233, 335803, 117044, and 370013) to M.H.V., M.L., and F.J.R.; the 10.13039/501100006306Sigrid Jusélius Foundation to M.H.V. and F.J.R.; the Finnish MS Foundation (Suomen MS-Säätiö) to M.H.V., T.K.K., and F.J.R.; 10.13039/501100020884Agencia Nacional de Investigación y Desarrollo (ANID, Chile)-FONDECYT Program Regular grant no. 1201706 to F.J.R., and the ANID-National Doctoral Fellowship no. 21211727 to C.R.R. In addition, the authors thank the PROFI 6 N° 336234 of the Research Council of Finland. The graphical abstract was created in BioRender (https://BioRender.com/nr3gx9h).

## Author contributions

Conceptualization, T.K.K. and M.H.V.; methodology, T.K.K., C.R.R., A.S., J.N., M.L., and L.B.; investigation, T.K.K., C.R.R., J.N., S.M., A.S., A.M., L.B., and T.A.E.K.; visualization, T.K.K., C.R.R., S.M., and T.A.E.K.; funding acquisition, M.H.V. and F.J.R.; supervision, M.H.V. and F.J.R.; writing – original draft, T.K.K.; writing – review & editing, T.K.K., C.R.R., J.N., A.S., S.M., L.B., A.M., T.A.E.K., M.L., F.J.R., and M.H.V.

## Declaration of interests

M.H.V. is a shareholder in MyNeuroCure Oy, which holds intellectual property rights related to medical applications of MANF peptides.

## Supplemental information

Data S1. Table S3

Document S2. Article plus supplemental information
