# Supplementary material for: Modulation of the unfolded protein response with a C-terminal fragment of MANF facilitates recovery in models of multiple sclerosis
Source: Mol Ther. 2025 Oct 11;34(2):1234–57. doi: 10.1016/j.ymthe.2025.10.023 (PMC12882342; doi:10.1016/j.ymthe.2025.10.023)
Supplement: Document S1. Figures S1–S7 and Tables S1 and S2 [file mmc1.pdf]

YMTHE, Volume *34*

Supplemental Information

Modulation of the unfolded protein response with a C-terminal fragment of MANF facilitates recovery in models of multiple sclerosis

Tapani K. Koppinen, Carolina R. Reyes, Jinhan Nam, Aastha Singh, Shibajee Mandal, Liam Beckett, Alba Montedeoca, Tuomas A.E. Kallionpää, Maria Lindahl, Francisco J. Rivera, and Merja H. Voutilainen

Supplemental Figures

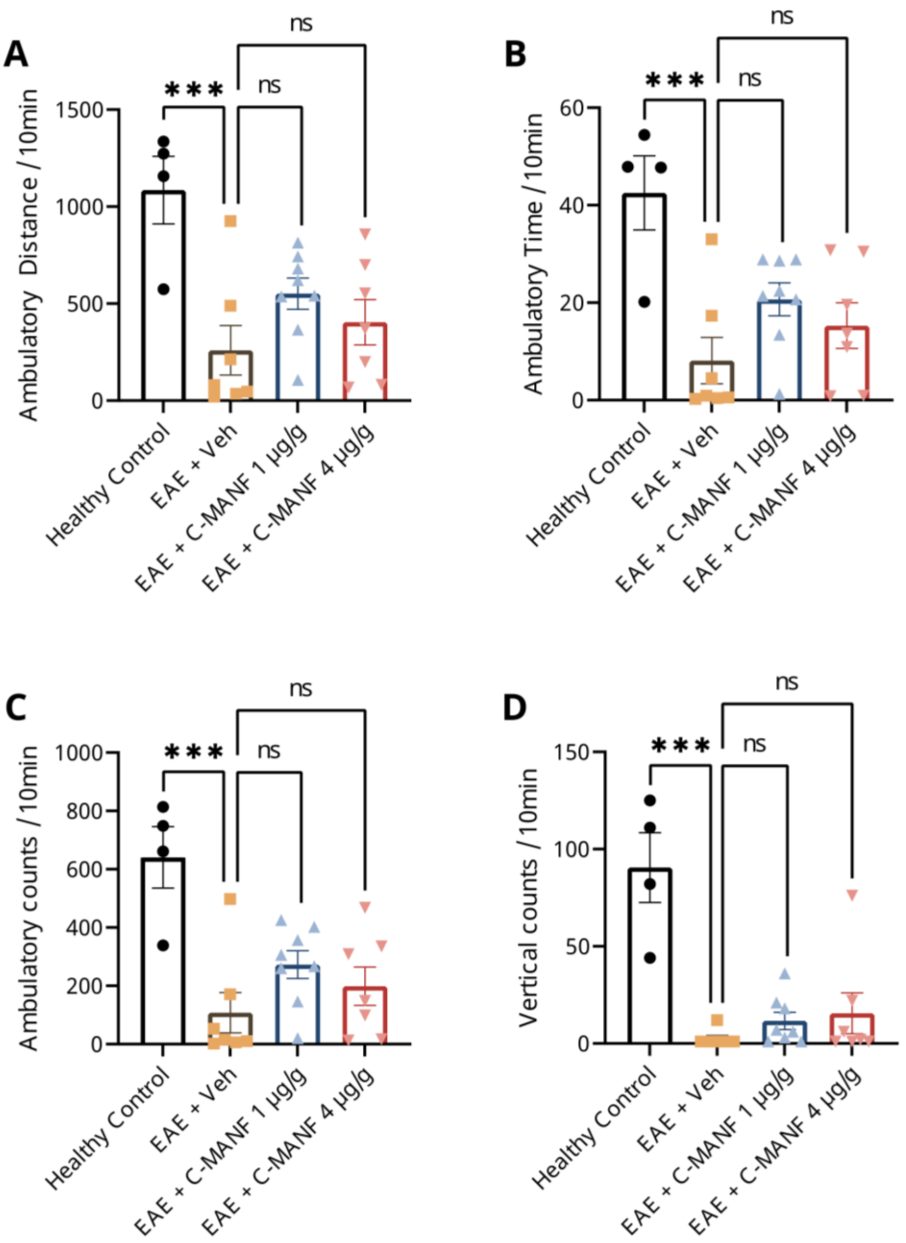

Figure S1. Effect of C-MANF administration on EAE-suppressed ambulation

Shown is ambulatory (**A**) distance, (**B**) time and (**C**) counts, as well as (**D**) vertical counts, performed by mice in 10 minutes of open field testing before sacrifice at day 28 of EAE. Each dot corresponds to a single animal, and bar graphs represent group means ± SEM, * $P \leq 0.05$. (A-D) Kruskal-Wallis test followed by Dunn's post hoc test.

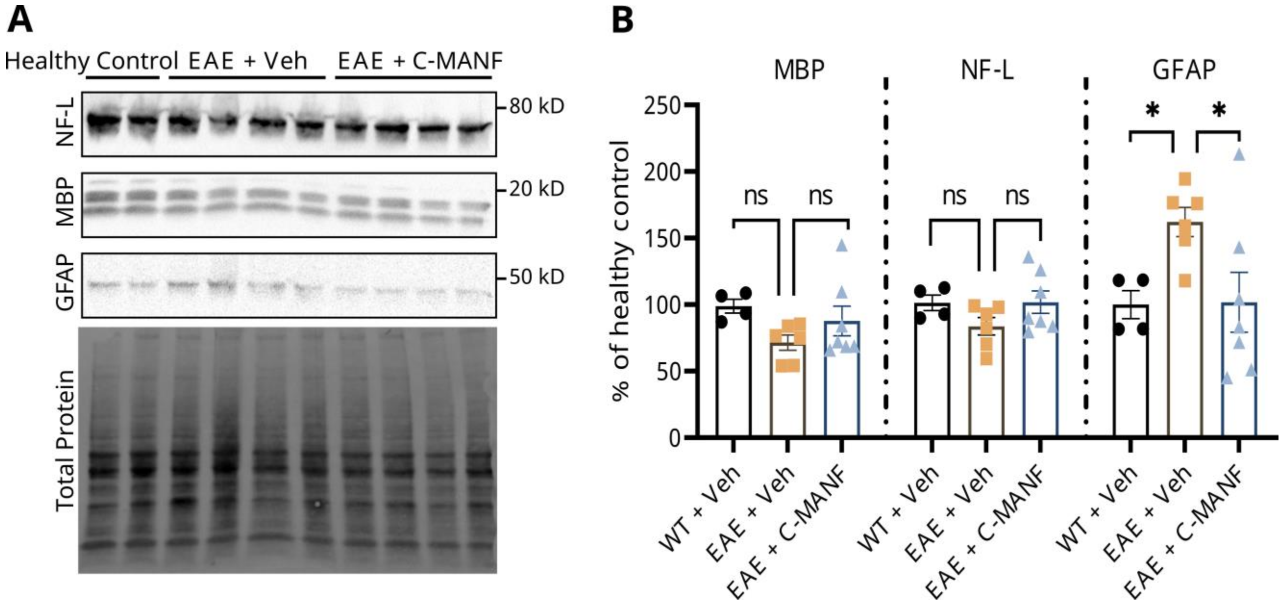

Figure S2. Effect of C-MANF administration on myelin, axonal and astrocytic protein expression in EAE

(**A**) Representative western blots featuring protein extracted from spinal cords of healthy controls and vehicle or C-MANF-treated EAE mice, as well as sample-matched total protein visualized with Stain Free technology. Each sample/lane is from one mouse. (**B**) Densitometric analysis of MBP, neurofilament light chain (NF-L) and GFAP-expression from western blots (data combined from two blots), normalized to total protein and reported as % of average of healthy controls. Each dot represents protein extracted from a single mouse spinal cord. Bar graphs represent group means ± SEM, * $P \leq 0.05$. (B) One-way ANOVA followed by Šidak's post hoc test.

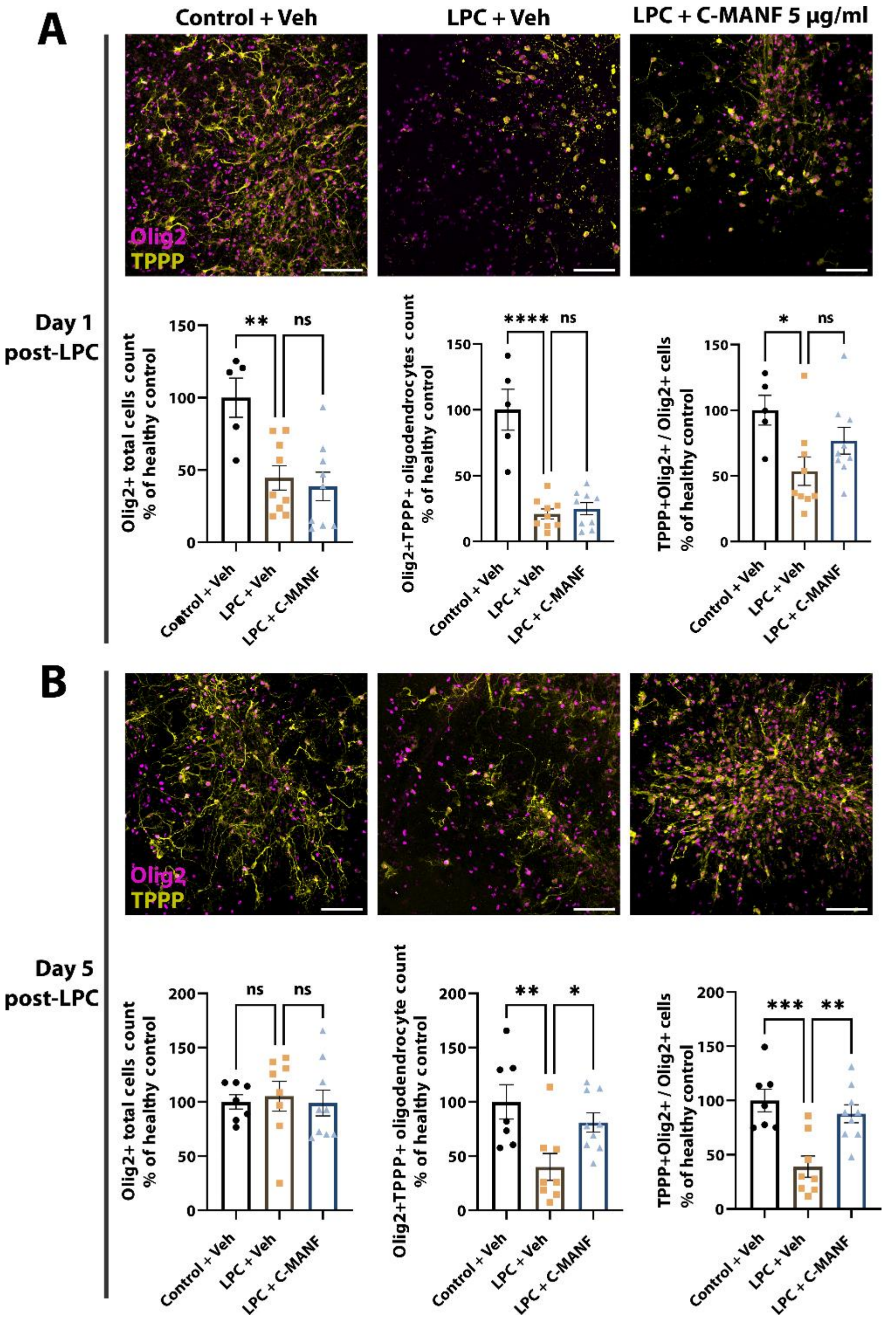

Figure S3. C-MANF improves oligodendrocyte differentiation ex vivo following demyelination with LPC

Representative 20x IF images of organotypic slices stained for Olig2 and TPPP, featuring naïve untreated slices treated with a vehicle as well as slices demyelinated for 17h with LPC and then treated with vehicle or C-MANF

for (**A**) 24h and (**B**) 5 days. The area fraction of Olig2+ cells and Olig2+TPPP+ cells, as well as the fraction of TPPP+Olig+ over Olig+ cells, is reported for both timepoints separately, each dot corresponding to a single well (average of 2 slices / well, 2 images / slice) and reported as % of average of naïve untreated control group. Scale bar = 100 µm. Bar graphs represent group means ± SEM, * $P \leq 0.05$, ** $P \leq 0.01$, *** $P \leq 0.001$ and **** $P \leq 0.0001$. (A-B) One-way ANOVA followed by Holm-Šidak's post hoc tests.

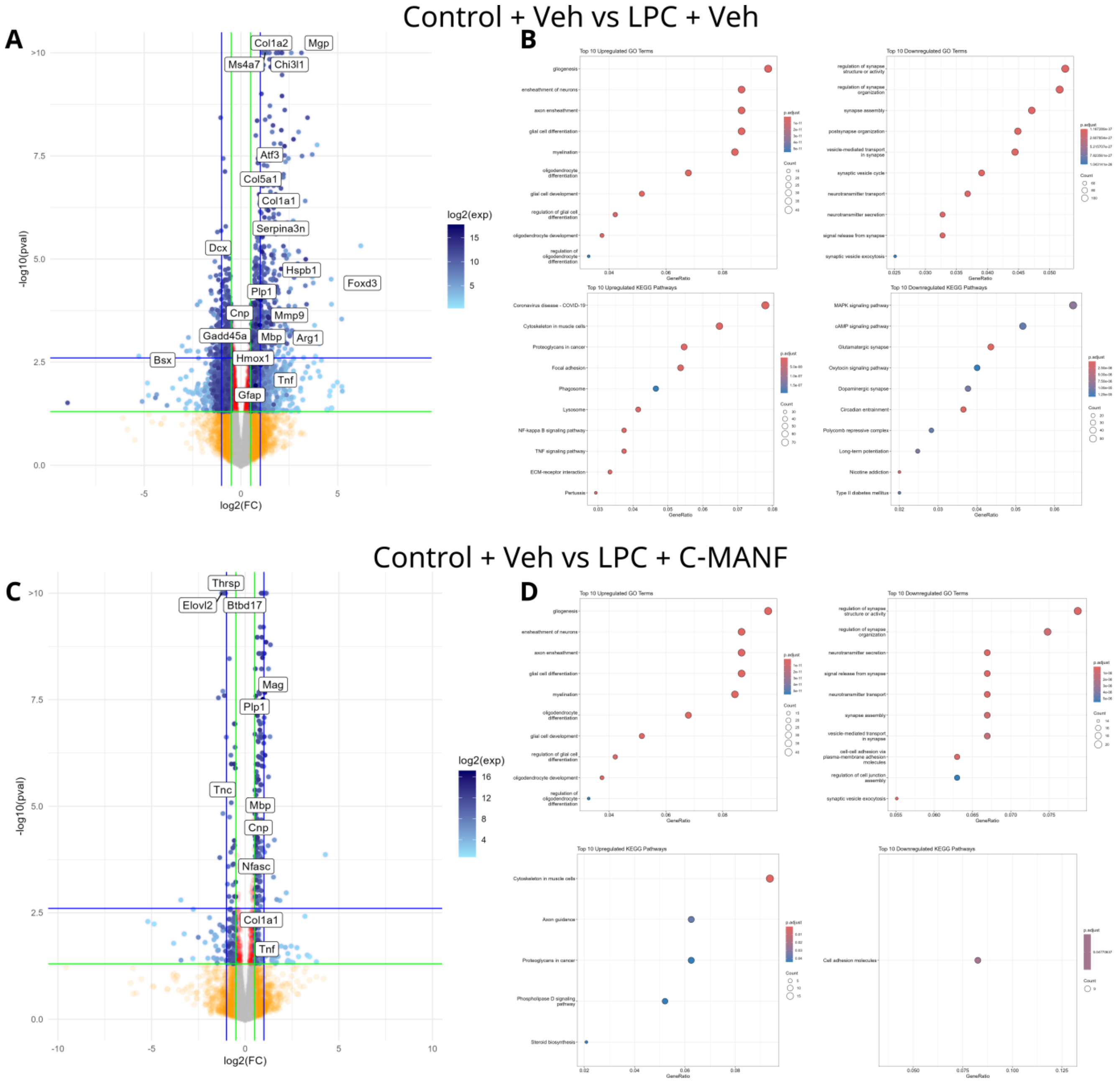

Figure S4. Significantly upregulated and downregulated genes and pathways between LPC-treated and Untreated organotypic slices

(**A**) Volcano plot of -log10 of adjusted P-values against log2-fold change. Blue dots indicate differentially expressed genes (DEGs) between Control + Vehicle vs LPC + Vehicle groups, with selected genes additionally labelled. (**B**) The 10 most significantly upregulated and downregulated GO (gene ontology) and KEGG terms between Control + Vehicle vs LPC + Vehicle groups. (**C**) Volcano plot of -log10 of adjusted P-values against log2-fold change. Blue dots indicate differentially expressed genes (DEGs) between Control + Vehicle vs LPC + C-MANF groups, with selected genes additionally labelled. (**D**) The 10 most significantly upregulated and downregulated GO and KEGG terms between Control + Vehicle vs LPC + C-MANF groups.

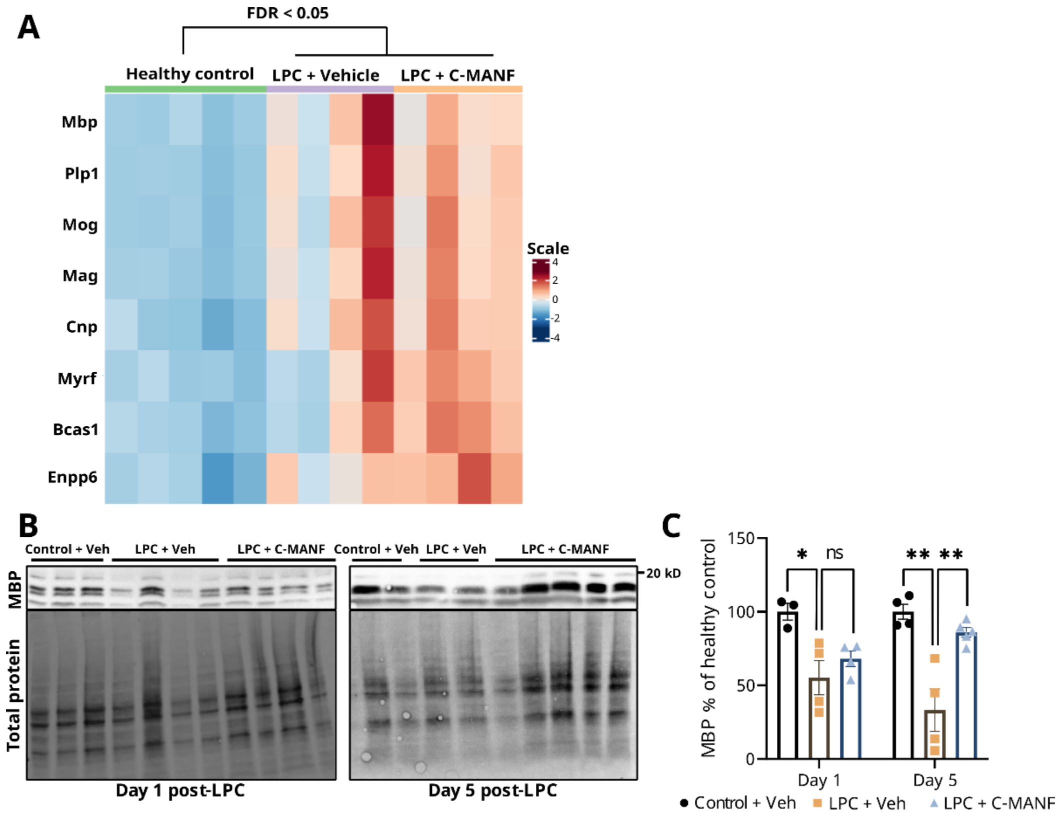

Figure S5. Effect of LPC and C-MANF administration on the expression of myelin genes and proteins

(**A**) Heatmap of individual sample expression of a selected panel of genes involved in myelination, grouped by their labelled roles and colored by z-score normalization. All shown genes are significantly upregulated between the healthy controls and both the LPC + Vehicle or LPC + C-MANF groups. (**B**) Representative western blots featuring of protein extracted from organotypic brain slices at 1 and 5 days post-LPC, as well as sample-matched total protein visualized with Stain Free technology. Each sample/lane was prepared by pooling together 4-5 slices grown in the same well. (**C**) Densitometric analysis of MBP-expression from western blots (data from three blots), normalized to total protein and reported as % of average of naïve untreated control. Bar graphs represent group means ± SEM, ** $P \leq 0.01$. (B) Two-way ANOVA, followed by Holm-Šidak's post hoc test.

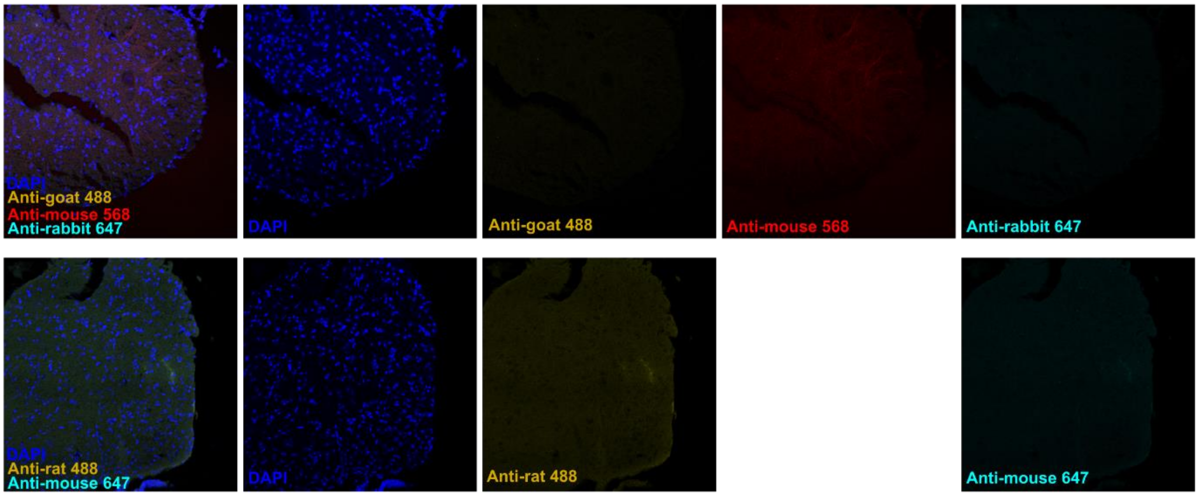

Figure S6. Secondary antibody controls for antibodies used for IF

Shown are 20x IF images of mouse lumbar spinal cords stained using the secondary conjugated antibodies from Table S1 as previously described, but omitting any primary antibodies. Confocal micrographs taken at very high exposure settings.

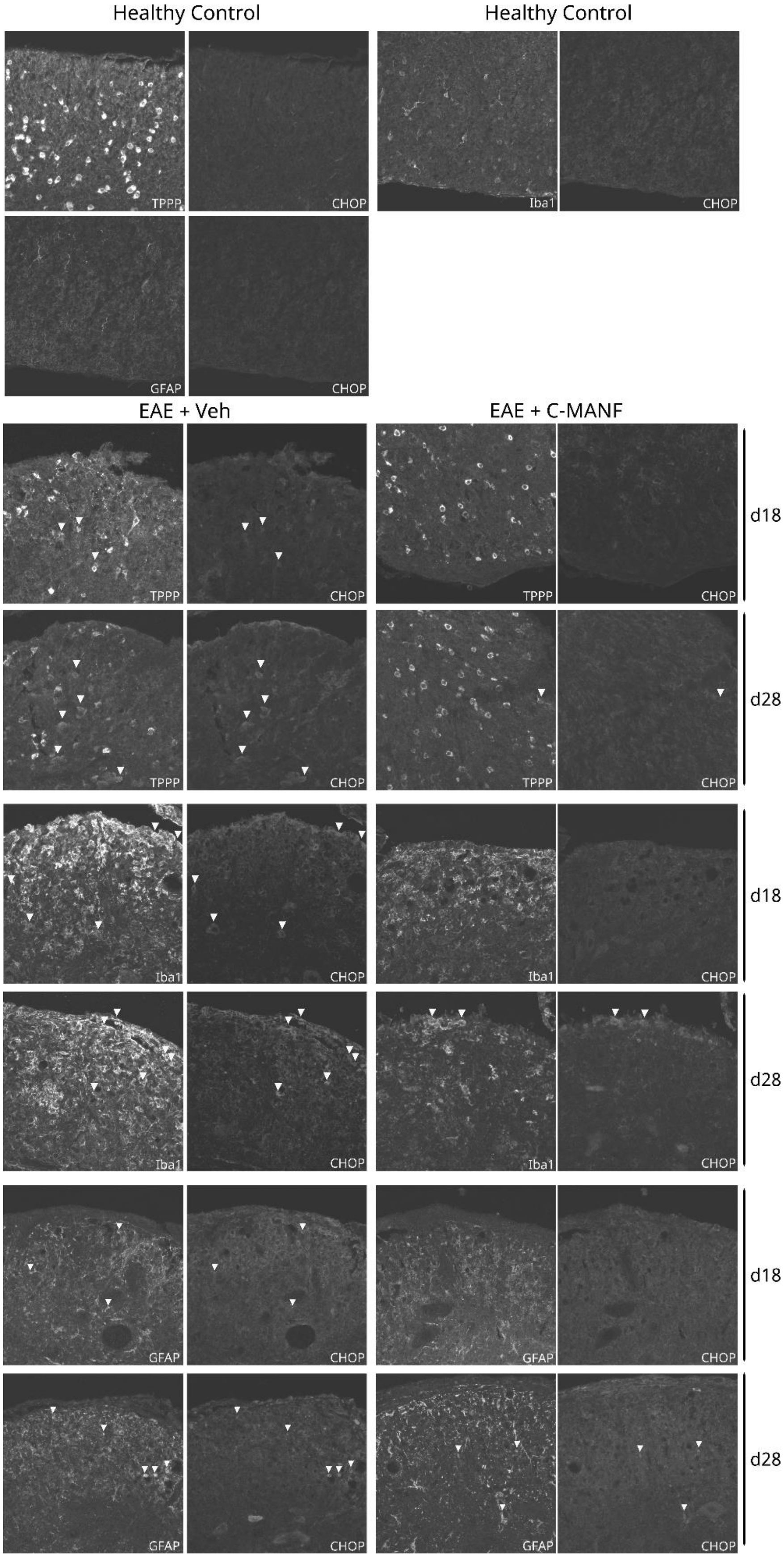
Healthy Control
Healthy Control
TPPP
CHOP
Iba1
CHOP
GFAP
CHOP
EAE + Veh
EAE + C-MANF
TPPP
CHOP
TPPP
CHOP
d18
TPPP
CHOP
TPPP
CHOP
d28
Iba1
CHOP
Iba1
CHOP
d18
Iba1
CHOP
Iba1
CHOP
d28
GFAP
CHOP
GFAP
CHOP
d18
GFAP
CHOP
GFAP
CHOP
d28

Figure S7. Individual channel images for Figure 3

Shown are individual grayscale images of the TPPP, Iba1, GFAP and CHOP fluorescent images from Figure 3. White arrows denote CHOP+ cells.

Supplemental Tables

Table S1: Primary Antibodies used in immunofluorescence, western blotting & flow cytometry analyses

Use	Antibody	Manufacturer	RRID	Used in Fig. X	Concentration
Primary unconjugated antibodies:	MBP	Santa Cruz	AB_10655672	Fig. 2, 5, 7, 8 & Fig. S5	1:500
	NF-200	Sigma	AB_477272	Fig. 2, 5, 7, 8	1:500
	TPPP	Thermo Fisher Scientific	AB_10979400	Fig. 3, 8 & Fig. S3	1:500
	CHOP	Santa Cruz	AB_627411	Fig. 3	1:250
	GFAP	Novus Biologicals	AB_829022	Fig. 3	1:500
	Iba1	Fujifilm Waco Chemicals	AB_2936184	Fig. 3 & 8	1:500
	CD3	Santa Cruz	AB_627014	Fig. 4	1:250
	MBP (Primary OPC)	Biorad	AB_325004	Fig. 5	1:500
	Olig2	Millipore	AB_570666	Fig. 5, Fig. S3	1:350
Secondary conjugated antibodies:	Donkey anti-goat 488	Invitrogen	AB_2534102	Fig. 3, 8 & Fig. S6	1:400
	Donkey anti-mouse 568	Invitrogen	AB_2534013	Fig. 2, 3, 5, 7, 8, Fig. S3 & Fig. S6	1:400
	Donkey anti-mouse 647	Invitrogen	AB_162542	Fig. 4 & Fig. S6	1:400
	Donkey anti-rabbit 647	Invitrogen	AB_2536183	Fig. 2, 3, 5, 7, 8, Fig. S3 & Fig. S6	1:400
	Donkey anti-rat 488	Invitrogen	AB_2536183	Fig. 5 & Fig. S6	1:500
Western blotting primary antibodies:	MBP	Santa Cruz	AB_10655672	Fig. 5, Fig. S2 & Fig. S4	1:1000
	NF-L	Cell Signaling Technology	AB_823575	Fig. S2	1:1000
	GFAP	Novus Biologicals	AB_829022	Fig. S2	1:1000
Flow Cytometry:	CD45 FITC	BD Biosciences	AB_395570	Fig. 4	1:500
	CD11b PE-Cy7	BD Biosciences	AB_394491	Fig. 4	1:200
	CD4 BV421	BD Biosciences	AB_396634	Fig. 4	1:200
	CD8a PerCP-Cy 5.5	BD Biosciences	AB_394081	Fig. 4	1:200

Table S2: Primer sequences used in RT-qPCR

**Gene target**	**Sequence**
Grp78	Forward: 5'-CTGGGTACATTTGATCTGACTGG-3'
	Reverse: 5'-GCATCCTGGTGGCTTTCCAGCCATTC-3'
Xbp1s	Forward: 5'-GAGTCCGCAGCAGGTG-3'
	Reverse: 5'-GTGTCAGAGTC-CATGGGA-3'
Atf4	Forward: 5'-CTGGGTACATTTGATCTGACTGG-3'
	Reverse: 5'-CGAAGTCAAACTCTTTCAGATCCATT-3'
Atf6	Forward: 5'-GGACGAGGTGGTGTCAGAG-3'
	Reverse: 5'-GACAGCTCTTCGCTTTGGAC-3'
Chop	Forward: 5'-CCAACAGAGGTCACACGCAC-3'
	Reverse: 5'-TGACTGGAATCTGGAGAGCGA-3'
Gfap	Forward: 5'-CAAGATGAAACCAACCTGAGGCT-3'
	Reverse: 5'-GGCTTGGCCACATCCATCT-3'
Cd11b	Forward: 5'-TACCGGAAGGATTCAGCAAG-3'
	Reverse: 5'-TAGCAGGAAAGATGGGATGG-3'
*Tnf*α	Forward: 5'-TGCCTCAGCCTCTTCTCATT-3'
	Reverse: 5'-CCCATTTGGGAACTTCTCCT-3'
Actin	Forward: 5'-GATCTGGCACCACACCTTCT--3'
	Reverse: 5'-GGGGTGTTGAAGGTCTCAAA-3'

Table S3: Lists of differentially expressed genes for the comparisons in Figure 6 and Figure S4
